# A randomized controlled trial of structured palliative care versus standard supportive care for patients enrolled in phase 1 clinical trials

**DOI:** 10.1002/cam4.3971

**Published:** 2021-05-25

**Authors:** Michelle Treasure, Barbara Daly, Shufen Cao, Pingfu Fu, Augustine Hong, Elizabeth Weinstein, Jessica Surdam, Neal J. Meropol, Afshin Dowlati

**Affiliations:** ^1^ Department of Medicine University Hospitals Cleveland Medical Center Seidman Cancer Center and Case Western Reserve University Cleveland OH USA; ^2^ Frances Payne School of Nursing Case Comprehensive Cancer Center Case Western Reserve University Cleveland OH USA; ^3^ Department of Population and Quantitative Health Sciences Case Western Reserve University Case Comprehensive Cancer Center Cleveland OH USA; ^4^ University Hospitals Cleveland Medical Center Seidman Cancer Center Case Comprehensive Cancer Center Case Western Reserve University Cleveland OH USA

## Abstract

**Purpose:**

Patients enrolled in Phase 1 clinical trials have typically exhausted standard therapies and often are choosing between a clinical trial and hospice care. Significant symptom burden can result in early trial discontinuation and confound trial outcomes. This study aimed to examine differences in study duration, symptom burden, adverse events (AE), and quality of life (QOL) between those receiving structured palliative care versus usual supportive care.

**Patients and methods:**

Sixty‐eight patients enrolled in phase 1 clinical trials and 39 of their CGs were randomly assigned to receive structured palliative care or usual supportive care. Patient QOL was measured monthly using the Functional Assessment of Cancer Therapy and Memorial Symptom Assessment Scale. The Quality of Life in Life‐Threatening Illness–Family Care Version and Caregiver Reaction Assessment were used for CGs. AEs and use of palliative care resources were compared between arms.

**Results:**

Mean duration of the phase 1 study was 142 days in the palliative care arm versus 116 days in the usual care arm (*p* = 0.55). Although not statistically significant, patients in the palliative care arm experienced fewer AEs and better QOL, as did their CGs, compared to those receiving usual care.

**Conclusions:**

Phase 1 patients and their CGs have physical and psychosocial needs warranting palliative care services. Results suggest that structured palliative care is associated with the increased duration of the study and improved patient and CG QOL.

## Abstract 2

Phase 1 patients and their caregivers have physical and psychosocial needs warranting palliative care services. Providing structured palliative care servies to this population was associated with increased duration on phase 1 study and improved patient and caregiver QOL.

## 1 INTRODUCTION

Phase 1 clinical trials play an extremely important role in drug development. Their main goals are to evaluate the safety profile of new agents or combinations of agents, determine dose‐limiting toxicities, and recommend a phase II dose and schedule. Phase 1 trials have historically reported low response rates that are in the 5–10% range and average life expectancy between 5 and 6.5 months.^1^, ^2^ The landscape of phase 1 trials is evolving with the molecular profiling of tumors and the use of targeted therapies. Recent data have demonstrated improved response rates, up to 2​0 percent, particularly when biomarker‐based inclusion criteria are employed.^3^ While the trend toward improved outcomes is encouraging, patients who are eligible for Phase 1 studies have usually exhausted standard therapies and are at a point in their disease trajectory where they may be choosing between experimental therapies and hospice care.

While patients who participate in phase 1 trials typically have a good performance status, they have been found to have a similar or greater symptom burden than cancer patients not participating in clinical trials.^4^ This has implications for patients and trial outcomes as the number of active symptoms correlates with how well experimental therapies are tolerated^5^ and the risk for developing serious drug‐related toxicities,^6^ and may contribute to early study discontinuation.^7^ This can complicate the assessment and attribution of symptoms and adverse events as to whether they are disease‐ or treatment‐related. Underappreciated emotional distress additionally may confound physical symptoms,^8^, ^9^ possibly influencing patient decision making regarding trial participation and potentially confusing the side effect profile with effects of the experimental agents on quality of life.

Phase 1 trials usually have the rigorous treatment and assessment schedules, and the patients who participate in these studies often have strong social support which enables them to participate. The informal caregivers of advanced cancer patients are increasingly recognized as an integral part of the patient's comprehensive care. The caregiving experience is complex, with the average amount of hours spent caregiving equating to a full‐time job.^10^, ^11^ Supporting the caregiver results in positive outcomes for caregivers and patients.^10^, ^12^, ^13^, ^14^, ^15^

Palliative care is an essential part of providing comprehensive care to patients with cancer and is recommended to be incorporated early into the care plans of patients with advanced cancer.^15^ Providing simultaneous palliative care services to patients enrolled in phase 1 clinical trials and their caregivers offer patients the opportunity to participate in research, with the benefits of aggressively managing symptom burden while addressing/transitioning goals of care in the context of a life‐limiting illness.

The primary purpose of this study was to assess symptom burden, adverse events, duration of the study, and quality of life among patients in phase 1 clinical trials who received structured palliative care and those who received standard care.

## 2 METHODS

### 2.1 Study design

This was a randomized clinical trial evaluating standard supportive care versus structured simultaneous palliative care in patients enrolled in phase 1 clinical trials. A contemporaneous, observational cohort of patients already established with palliative care (n = 12) was not included in this analysis. Patient caregivers were also enrolled if interested and randomized to the same arm as the patient. The study was approved by the IRB at Seidman Cancer Center, University Hospitals Cleveland Medical Center.

### 2.2 Participants

All patients with solid tumors, ages 18 and older, enrolled in a therapeutic solid tumor phase 1 clinical trial were considered eligible and approached for informed consent. Patients were asked to identify their caregiver, defined as the person who most often helps them, and caregivers were also approached and offered enrollment. All identified, unpaid caregivers were considered eligible.

### 2.3 Intervention

Patients in the standard supportive care arm received supportive care from their treating oncologists. The frequency of visits and referral to other specialties or services were at the discretion of the treating oncologist. The support for caregivers in the standard supportive care arm was also provided by the treating oncology team and referral to psychosocial personnel was at their discretion.

In the structured palliative care cohort, supportive care was provided by an outpatient palliative care team. The team consisted of clinicians with specialized palliative care training, social workers, spiritual care specialists, and mental health clinical nurse specialists (CNS). Each patient was required to have an initial, comprehensive medical, physical, and psychosocial evaluation with a palliative care physician or advanced practice nurse (APN) within 2 weeks of enrollment onto the phase I clinical trial. The patient was then to meet in person, at least monthly (30 +/‐ 7 days) until removed from the phase I clinical trial. Patients completed the Memorial Symptom Assessment Scale – Short Form (MSAS‐SF) before their visits and the results were used to guide symptom assessment. Patients could be seen more frequently if needed. The palliative care provider also periodically addressed goals of care and advance care planning. Caregivers in this group communicated in person or by phone with a psychosocial provider (social worker, mental health CNS, spiritual care provider) within 3 weeks of patient enrollment and then monthly either in person or by phone until the patient completed the phase 1 study.

### 2.4 Measures

Demographic information included age, gender, race, ethnicity, family income, and reason for enrollment onto phase 1 clinical trial. Other baseline data, included tumor type, stage, number of prior therapies, and ECOG performance status. Demographic information for the caregiver was self‐reported at baseline and included age, relationship to the patient, gender, race, ethnicity, education level, employment status, and other caregiving responsibilities at home.

Patients in each cohort were required to complete the Memorial Symptom Assessment scale short form (MSAS‐SF) as well as the Functional Assessment of Cancer Therapy –General (FACT‐G) at baseline and then monthly while enrolled on phase 1 clinical trial.

The MSAS‐SF is a self‐administered questionnaire that asks respondents to rate 28 physical and 4 psychological cancer‐related symptoms. Patients are asked whether they experienced a symptom within the past 7 days (yes/no), and if experienced how much distress the symptom caused using a 5‐point Likert scale. Higher scores indicated increased symptom burden.

The FACT‐G is a self‐administered 27 item questionnaire with 4 health‐related quality of life domains (physical, social/family, emotional, and functional). Patients are asked how they have felt over the last week, and questions are answered on a 5‐point Likert scale ranging from 0 = not at all to 4 = very much. The answers are summed for a total score, with higher scores indicating better quality of life.

Caregivers in each cohort were required to complete the Caregiver Reaction Assessment (CRA) and the Quality of Life in Life‐Threatening illness‐Family Carer Version (QOLLTI‐F), at baseline and then monthly while the patient was enrolled. The CRA is a self‐administered 24 item questionnaire addressing five domains: self‐esteem, family support, finances, daily schedule, and health. The perceived impact is rated on a 5‐point Likert scale, from 1 = strongly disagree to 5 = strongly agree. Each subscale is totaled and divided by the number of items to reflect an unweighted mean‐item score with a range from 1 to 5. Higher scores on all subscales indicate negative experience, except for self‐esteem where a high score indicated a positive experience. Grove and Colleagues recommend reverse coding the self‐esteem score so that the total CRA score reflects the overall caregiver situation with higher scores indicating higher caregiver burden.

The QOLLTI‐F is a self‐administered 16 item questionnaire used to measure family caregiver quality of life over the previous 2 days, particularly when caring for someone with a life‐threatening illness. The questions cover seven domains including environment, patient condition, quality of care, carer's outlook, carer's state, and financial worries. The response scale is an 11 point numerical rating scale that ranges from 0 to 10, with 10 being the best possible situation, with higher scores indicating better quality of life.

We also evaluated each participant's adverse events profile, which was collected by the phase 1 clinical trial team, and used the CTEP Common Terminology Criteria for Adverse Events (CTCAEv4). In addition to the raw number of events, we used an exploratory weighted adverse event score based on the number of adverse events as well as the adverse event grade. Weighted adverse event score = ⅀(N_AE_ x G_AE_); N denotes the adverse event number and G denotes grade and AE denotes adverse event. To account for varying durations on trial potentially contributing to a number of adverse events, we also calculated a “rate” by dividing the number of AEs by the days on trial. We did this for both raw and weighted adverse events.

### 2.5 Statistical analysis

An estimated sample size of 76 was determined by the hypothesis that structured palliative care would increase the duration of phase 1 trial by at least 30 days. By randomized phase II screening design,^16^ a two group one‐sided T‐test with a 0.2 significance level had 80 percent power to detect a difference of 40 days (effect size = 0.388) on trial between arms.

The difference in continuous measurements (age, number of therapy, baseline assessment of various types of symptoms‐ total and subscales and duration on treatment) between the two treatment arms was examined using T‐Tests. The associations among categorical variables were examined using chi‐square test. The effects of age, gender, race, income, caregiver (yes/no), performance status, and age on total Fact G were estimated using multivariable regression, as were the effects of age, gender, race, education, income, type of caregiver, employment, and other responsibility at home on total QOLLTIF and CRA.

The scores of CRA, MSAS, FACT‐G, and QOLLTIF were examined by T‐test at day 32+/‐ 8, 61 +/‐ 8, and 88 +/‐ 8. The temporal profiles of those longitudinal assessments were visualized using scatter plots superimposed with lowess (locally weighted scatterplot smoothing) smoother. In order to estimate the “growth” rate of those assessments over time, a mixed model approach was used. In mixed longitudinal models, the assumption is that measurements during follow‐up from the same individual are correlated and unstructured covariance was used for inference. All tests were two‐sided and p values less than 0.05 were considered statistically significant.

## 3 RESULTS

### 3.1 Patient results

**FIGURE 1:**
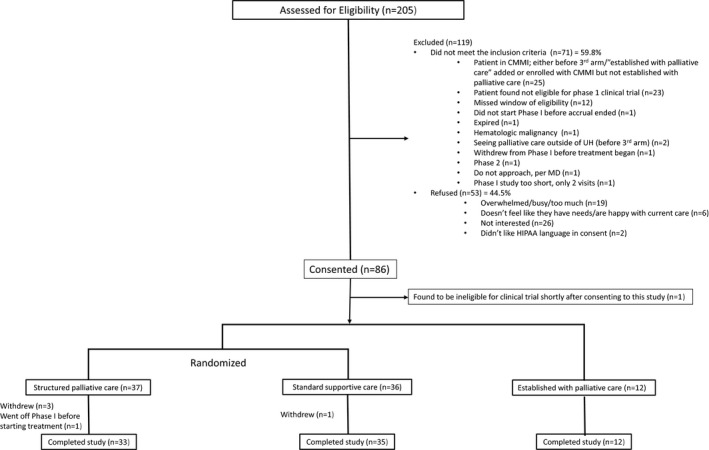
Patient consort diagram

Eighty‐five eligible patients enrolled in the study and 80 patients completed the study. Seventy‐three participants (85.8%) were randomized to receive either structured palliative care (n = 37) or standard care (n = 36), and 12 participants (14.1%) were already established with palliative care and were not included in this analysis (Figure 1).

#### 3.1.1 Demographics

Baseline patient characteristics are summarized in Table 1. There were no statistically significant differences in baseline characteristics between arms.

**TABLE 1:** Patient characteristics

Factor	frequency (%)
Age (year): median (range)	62 (35, 91)
# of prior therapy: median (range)	2.2 (2.11)
Gender (Male /Female)	35 (51.47)/ 33 (48.53)
Race (Asian /Black /White)	2 (2.94) / 7 (10.29) / 59 (86.76)
Financial Income	
< 20 k	11 (16.18)
20 k – 50 k	24 (35.29)
≥ 50 k	30 (44.12)
Diagnosis	
Lung cancer	17 (25.00)
Breast cancer	3 (4.41)
GI cancer	22 (32.35)
GU cancer	7 (10.29)
Gynecologic cancer	7 (10.29)
Melanoma	3 (4.41)
CNS tumor	5 (7.35)
Other	4 (5.88)
PS (0/1/2/3)	30 (44.78)/ 36 (53.73) /0/ 1 (1.49)
Reason to enroll	
Help future patients	16 (23.53)
Family wanted	4 (5.88)
To feel better	6 (8.82)
Hope for cure	19 (27.94)
Hope for other medical benefits	7 (10.29)
Trust in the doctor who recommended	21 (30.88)
No better option	8 (11.76)
Other	9 (13.24)
PS at time of discontinuation	
0/1/2/3	18 (33.96) /28 (52.83) /5 (9.42) /2 (3.77)

#### 3.1.2 Duration of the study, symptom burden, and quality of life

Although not statistically significant, average point estimates showed patients in the structured palliative care arm remained on the study 26 days longer than those in the standard arm (142 days versus 116 days; *p* = 0.55).

All patients had some degree of baseline adverse events or symptom burden. There were no notable differences between arms in regards to baseline symptom (MSAS‐SF) or quality of life (FACT‐G) measures other than the slightly better quality of life scores in the domain of social and family well‐being in the standard care arm (see Table 2). The average number of baseline physical symptoms and psychological symptoms reported through the MSAS‐SF was similar between groups with an average of 3.6 (STD 2.7) physical symptoms and 2.1 (STD 2.0) psychological symptoms.

**TABLE 2:** Baseline assessment of MSAS, FACT‐G, QOLLTIF, and CRA

Type of symptoms	mean (STD)
**Total MSAS (sum of 30 items)**	**21.73 (14.93** **)**
PHYS (sum of 11 items)	10.76 (8.17)
PSYCH (sum of 6 items)	5.61 (5.31)
GDI (sum of 10 items)	11.55 (8.41)
**Total FACT‐G** (sum of 27 items)	**77.65 (17.29)**
Physical well‐being (sum of 7 items)	21.78 (5.54)
Social/family well‐being (sum of 7 items)	21.56 (6.90)
Emotional well‐being (sum of 6 items)	18.46 (4.64)
Functional well‐being (sum of 7 items)	18.67 (6.57)
**Total QOLLTIF** (sum of 16 items)	**127.87 (21.00)**
Environment	16.26 (4.32)
Patient condition	5.74 (3.61)
Own condition	39.61 (8.76)
Outlook	25.76 (4.08)
Quality of care	18.18 (2.50)
Relationship	14.84 (5.54)
Financial worries	7.47 (3.39)
**Total CRA** (sum of 24 items)	**50.89 (11.05)**
Self‐esteem	11.81 (2.60)
Lack of family support	8.84 (3.82)
Impact of finances	6.46 (2.85)
Impact of the daily schedule	15.32 (4.43)
Impact of health	8.61 (2.99)

The bold text indicate the total scores of quality of life assessment tools we used to assess both the patient/phase 1 participant symptom burden and quality of life and the caregiver burden and quality of life.

The adverse events reported through each phase 1 study were compared between groups and there was no statistically significant difference in the number of baseline reported events (prior to starting investigational agent), which averaged 13.8 (STD 8.2); the average weighted adverse event score, which accounted for the grade was 16.72 (STD 11.61). We classified the adverse events into laboratory and non‐laboratory adverse events. The average number of baseline non‐laboratory events was 9.7 (STD 6.1) and the average number of weighted non‐lab adverse events was 11.53 (STD 7.31). There was a moderate correlation between the baseline total MSAS‐SF scores and the baseline weighted (r = 0.4, *p* = 0.0004) and non‐weighted (r = 0.27, *p* = 0.018) non‐laboratory adverse events. As expected, the MSAS‐SF scores were inversely correlated with FACT–G scores, indicating that higher symptom burden was associated with impaired quality of life (r = −0.74; *p* ≤ 0.001).

**FIGURE 2:**
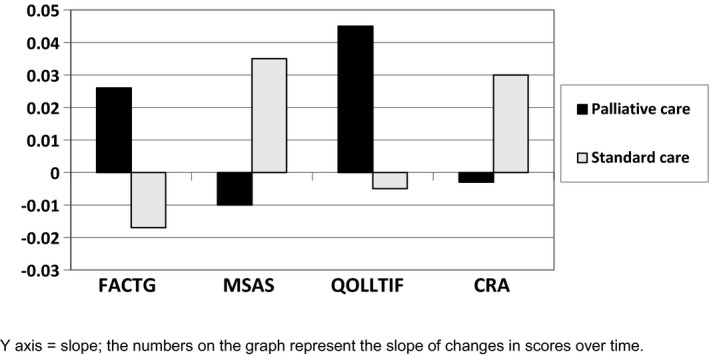
Change of symptom burden and quality of life over time (90 days) in patients and caregivers

Patient baseline QOL assessed through the FACT‐G was not influenced by age, race, income, or presence of the caregiver. However, gender was associated with total FACT‐G scores. Compared to females, the total mean FACT‐G score was 14.5 lower for males (*p* = 0.005). As with MSAS, we used the slope of the change in the total FACT‐G score to depict a change in QOL throughout the study, with higher scores indicating better outcomes (see Figure 2). While the differences between arms were not statistically significant, the trend favored the structured palliative care arm.

**FIGURE 3:**
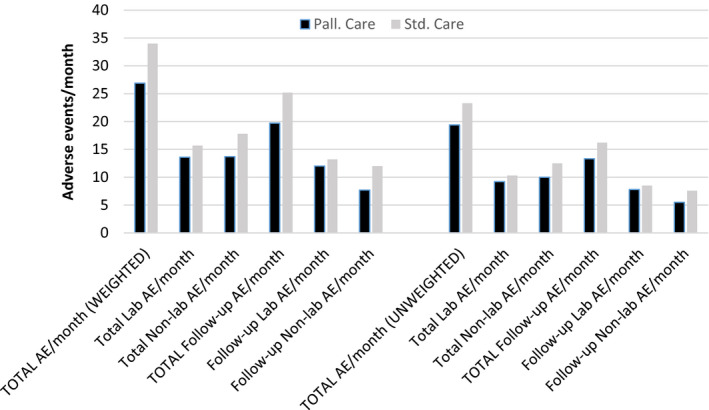
Comparison of adverse event rates (weighted and unweighted per month)

Patients in the structured palliative care arm also experienced fewer weighted and unweighted adverse events over time (adverse event rate), than those in the standard arm. As with QOL measures, the differences were not significant. (Figure 3).

While all subscales of the MSAS‐SF were evaluated over the course of the study, we used the change in the total MSAS‐SF scores to depict changes in symptom burden over time, with higher scores indicating increased symptom burden. In the structured palliative care arm, patient symptom burden decreased over time, while it increased in the standard care arm (see Figure 2).

Using the MSAS‐SF, the most frequent symptoms experienced were itching (100%), mouth sores (77.94), difficulty swallowing (72.06), vomiting (69.12), and hair loss (67.65). The most distressing symptoms reported were problems with sexual interest or activity, pain, lack of energy, lack of appetite, and difficulty sleeping.

#### 3.1.3 Services utilized

Patients in the structured palliative care arm had an average of 4.58 visits with a member of the palliative care team. In the standard arm, two patients were referred to palliative care services. Of all encounters in the structured palliative care arm, 17% of visits were with the physician, 65% were with an advanced practice nurse (APN), 13% with a social worker, 4% with spiritual care provider, and 1% with a mental health specialist. Services provided are shown in Table 3.

**TABLE 3:** In‐person services provided to patients in structured palliative care

Services provided	Number of patients who had specific services (n = 33) (frequency (%))	Number of specific services out of all services (total visits = 201) (frequency(%))
Symptom assessment/management	33 (100)	143 (71.14)
Psychosocial issues	32 (96.97)	96 (47.76)
Spiritual care	24 (72.73)	50 (24.88)
Other	23 (69.70)	34 (16.92)
Advanced care planning	22 (66.67)	53 (26.37)
Coping	16 (48.48)	47 (23.38)
Referral to other specialties	16 (48.48)	22(10.95)

We assessed various measures of aggressiveness of care including antineoplastic treatments in the last 14 to 30 days of life, emergency room visits, hospitalizations, ICU stays, and length of time in hospice and found no difference between arms.

### 3.2 Caregiver results

**FIGURE 4:**
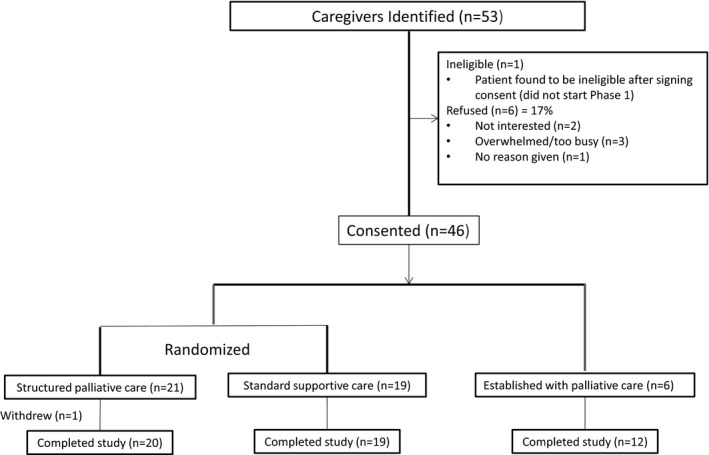
Caregiver consort diagram

Forty‐six caregivers (54.1% of the patient sample) enrolled and completed the study, and 39 caregivers were included in the analysis (Figure 4).

#### 3.2.1 Demographics

Baseline caregiver characteristics are summarized in Table 4. There were no statistically significant differences in baseline characteristics between arms. The majority of caregivers were spouses (51%), approximately 51% of caregivers were employed and 26% had other responsibilities at home.

**TABLE 4:** Caregiver characteristics

Factor	frequency (%)
Caregiver	
Spouse	20 (51.28)
Partner	4 (10.26)
Sibling	1 (2.56)
Parent	0 (0.00)
Friend	4 (10.26)
Child	10 (25.64)
Age (year): median (range)	58 (25, 83)
Gender (Male/Female)	12 (30.77)/ 27 (69.23)
Race (Asian/Black/White)	1 (2.56)/ 6 (15.38)/ 32 (82.05)
Employment (employed/unemployed)	20 (51.28)/ 19 (48.72)
Education	
< high school	1 (2.56)
High school	11 (28.21)
College (no degree)	9 (23.08)
Associate's degree	1 (2.56)
Bachelor	9 (23.08)
Post graduate	8 (20.51)
Other responsibility at home	
No	29 (74.36)
Yes	10 (25.64)

#### 3.2.2 Symptom burden and quality of life

All caregivers experienced some degree of baseline burden. There were no notable differences between arms in regards to baseline caregiver burden (CRA) or quality of life (QOLLTIF) (Table 2). The highest burden was seen on the impact on the daily schedule scale, followed by the impact on self‐esteem. Caregiver baseline symptom burden and QOL assessed through the CRA and QOLLTIF were not influenced by caregiver type, age, gender, race, employment, education, or other home responsibilities. The CRA scores were inversely correlated with QOLLTIF scores, with higher caregiver burden associated with greater impairment in quality of life (r = −0.60, *p* = 0.0001).

All of the correlations between patient and caregiver metrics were in the anticipated direction, though not all were statistically significant. Statistically significant correlations were seen between patient's psychologic distress and caregiver QOL (r = −0.41, *p* = 0.01), and between patient global distress index and caregiver quality of life (r = −0.34, *p* = 0.04), with higher distress in these areas associated with worse caregiver total quality of life. There was also a significant positive correlation between the patient's functional status as assessed through the FACT‐G and the caregiver quality of life (r = 0.47, *p* = 0.004).

Changes in total CRA scores were used to depict changes in caregiver burden over time, with higher scores indicating increased caregiver burden. In both arms, the total CRA scores decreased over the first month, then increased over the second month, and while not significant, caregiver burden was lower in the palliative care arm over time (Figure 2).

A similar approach was used to examine the change in the total QOLLTIF score depicting caregiver QOL, with higher scores indicating better outcomes. The QOL for caregivers improved in both arms, but was more notable in the structured palliative care arm (Figure 2), with a non‐significant trend favoring the structured palliative care arm.

## 4 DISCUSSION

Phase 1 oncology trials and palliative care have changed dramatically over the last decade. While our understanding of tumor biology and the advancement of molecularly targeted therapeutics has increased the promise of early phase studies, the primary purpose of phase 1 trials remains to assess toxicity rather than the efficacy of investigational agents. Patients who participate in these trials typically have advanced disease and have exhausted standard therapies, making them eligible for either investigational studies or hospice care. Most phase 1 participants also have significant baseline symptom burden or sequelae from their disease and prior therapies,^4^, ^17^ and phase 1 trials may increase symptoms and decrease quality of life.^18^ Simultaneously, the benefits of palliative care in patients with advanced cancer have been increasingly recognized, with improved clinical outcomes,^15^, ^18^, ^19^, ^20^ and it is recommended that palliative care be incorporated for all patients with advanced disease.^15^

Our data confirm that patients and caregivers have significant psychosocial needs in addition to physical symptoms and caregiving burden at the time of enrollment that warrants palliative care involvement.^4^, ^17^, ^19^, ^21^ We demonstrated that palliative care provided simulatenously to phase I trial enrollment is feasible, did not seem to add extra burden to patients or their caregivers, and potentially influenced duration on the study and positively affected QOL. All patients in the palliative care arm had symptoms requiring management at some point, nearly all patients had psychosocial issues, nearly 75% of patients had spiritual care needs, and approximately 50% of patients had issues coping and had symptoms that required referral to other specialties. Advance care planning was addressed with 67% of patients and 26% of all palliative care visits addressed advance care planning.

While Phase 1 patients are generally required to have good functional status (performance status) despite significant symptom burden at the time of enrollment, they have advanced disease with limited treatment options and their clinical status can deteriorate quickly. Addressing psychosocial needs and goals of care early, perhaps even during the informed consent process for the trial, is clearly indicated. Advance care planning is routinely incorporated as part of palliative care and can be an added benefit in future transitions.

Patient participation in phase 1 clinical trials and involvement in palliative care does not need to be mutually exclusive, and in fact, may be synergistic. In our study, patients in the structured palliative care arm remained on the study an average of 26 days longer than those in the standard supportive care arm. Though not statistically significant, this finding should be viewed as hypothesis‐generating and warrants further study given the average duration on phase 1 trials is between 1 and 3 months.^7^ Remaining on study nearly 4 weeks longer could enable 1–2 cycles of therapy, depending on the treatment regimen. The goal of palliative care is to maintain or improve patient function in addition to the quality of life, which benefits patients and may enable patients to stay on study longer or complete the trial, thus providing more data on investigational agents, their pharmacokinetics, and adverse event profiles, and contributing to the advancement of cancer therapeutics. Further, patients in the structured supportive care arm showed a trend to less adverse events over time compared to those in the standard supportive care arm. This may have implications for the toxicity profiles of investigational agents, as it is often difficult to determine whether an adverse event is related to the treatment or the disease itself, potentially confounding the assessment of adverse events and resulting in inaccurate toxicity profiles.

Patient‐reported outcomes are a pertinent topic in cancer therapeutics, augmenting the clinician‐based adverse event reporting system. In 2016, the NCI released a patient‐reported outcomes version of CTCAE,^22^ taking into account the patient perspective. We found patient‐reported symptoms through the MSAS‐SF strongly correlated with the study reported adverse events and the calculated weighted adverse event score. The weighted adverse event score may better depict the patient experience as it takes into account the number and grade of adverse events. In addition to the MSAS‐SF used in this study, there are several validated tools used by palliative care specialists, which are easily and regularly administered to patients to aid in symptom management. There is evidence that the assessment and management of patient‐reported symptoms lead to important benefits including improving quality of life and even survival.^23^

While not statistically significant, patient‐reported symptom burden and quality of life appeared to improve to a greater degree in the structured palliative care arm as compared to the standard supportive care arm. Our small sample size may have contributed to the lack of statistical significance, and our findings are consistent with the recently reported results of larger randomized controlled trial conducted by Smith et al, which showed improved quality of life outcomes and distress in phase 1 patients who received a palliative care intervention versus usual care.^24^

There was a strong correlation between patient QOL metrics and caregiver QOL metrics, supporting prior data on the reciprocal nature of the relationship.^10^, ^12^, ^25^ It is possible that through our structured support intervention, the caregiver better supported the patient, possibly contributing to the improvement in symptom and QOL metrics. Caregiver burden has not only been associated with decreased qualify of life of the caregiver but linked to other health issues as well including cardiovascular disease, depression, and early death.^10^, ^14^ Helping the caregiver transition goals of care with the patient has implications not only for improving their bereavement process and QOL, but also their overall health.^10^, ^14^, ^15^

While the involvement of early palliative care has been shown to decrease aggressive care measures near the end of life such as emergency room visits, hospitalizations, and ICU stays,^26^ we did not see a difference in these outcomes, nor other outcomes such as treatment in the last 14 or 30 days of life or length of hospice enrollment between arms. This may in part be secondary to our small sample size, but also the population of patients who choose to participate in phase 1 clinical trials is likely different than the average patient with advanced cancer. These patients have chosen to pursue aggressive therapy over best supportive care and therefore may be more likely to consent to additional interventions in the last several weeks of their life.

Regardless of the goals of care or treatment, structured palliative care and aggressive management of symptoms can support patients on phase 1 clinical trials, and potentially enable patients to stay on study longer. Thus, these interventions can both improve the quality of life for patients facing life‐limiting disease while advancing the development of new cancer therapeutics.

## CONFLICTS OF INTEREST

All authors have completed the ICMJE uniform disclosure form. Only Neal Meropol reported disclosure of others from Flatiron Health and Roche.

## Supporting information

Supplementary Material

## Data Availability

The data that support the findings of this study are available from the corresponding author upon reasonable request.
