# Supplementary material for: A randomized controlled trial of structured palliative care versus standard supportive care for patients enrolled in phase 1 clinical trials
Source: Cancer Med. 2021 May 25;10(13):4312–21. doi: 10.1002/cam4.3971 (PMC8267138; doi:10.1002/cam4.3971)
Supplement: Supplementary file 1 — Supplementary Material [file CAM4-10-4312-s001.pdf]

A Pilot Study of Structured Palliative Care for Patients Enrolled on Phase I Clinical Trials

Case Comprehensive Cancer Center

11100 Euclid Avenue
Cleveland, Ohio 44106-5065

CASE COMPREHENSIVE CANCER CENTER

STUDY NUMBER: CASE 5Y15

STUDY TITLE: A Pilot Study of Structured Palliative Care for Patients Enrolled on Phase I Clinical Trials

PRINCIPAL INVESTIGATOR:

Michelle Treasure, MD
Clinical Instructor, Department of Medicine
Division of Hematology and Oncology
Metro Health Medical Center
Case Western Reserve University
Cleveland, OH 44109
216-778-3978
Mxt269@case.edu

CO-PI:

Barbra Daly PhD, RN, FAAN
Director, Clinical Ethics
Case Western Reserve University
University Hospitals Case Medical Center
11100 Euclid Avenue
Cleveland, OH 44106
Bjd4@case.edu

CO- INVESTIGATOR:

Neal J. Meropol, MD
Case Comprehensive Cancer Center
University Hospitals Case Medical Center
Seidman Cancer Center
11100 Euclid Avenue
Cleveland, OH 44106
216-844-5220
Neal.Meropol@uhhospitals.org

Elizabeth Weinstein MD, MS
Medical Director, Supportive Oncology
Case Comprehensive Cancer Center
University Hospitals Case Medical Center
Seidman Cancer Center
11100 Euclid Avenue
Cleveland, OH 44106
Elizabeth.weinstein@uhhospitals.org

Afshin Dowlati, M.D.
Department of Medicine
University Hospitals of Cleveland
Case Western Reserve University
11100 Euclid Avenue
Cleveland, OH 44106
Tel: 216-844-8573
Email: axd44@cwru.edu

Dale R. Shepard, MD, PhD, FACP
Director, Taussig Cancer Institute Phase I Program
Staff, Department of Hematology/Oncology and Center for Geriatric Medicine
Cleveland Clinic Taussig Cancer Institute
9500 Euclid Avenue/R35
Cleveland OH, 44195
Phone: 216-445-5670
Email: shepard@ccf.org

STATISTICIAN: Pingfu Fu Ph.D.
Biostatistics Core Facility
Case Western Reserve University

SPONSOR: Case Comprehensive Cancer Center

SUPPORT: Conquer Cancer Foundation of the American Society of Clinical Oncology

History of Amendments

Amendment #1/Version date: February 8, 2016	
Section #/page	Description of change (s)
	Added the Cleveland Clinic as a site (Dale Shepard, site PI)
**5.4.2.1**	Added specific information to be tracked in follow-up, and the source and process for obtaining the information
**3.2**	Changed exclusion criteria to include patients already established with palliative care for a 3rd arm in the study for comparison
**5.3.3**	Description of intervention for added 3rd arm
**6.0**	Added 3rd arm into statistical analysis section
**Appendices E&F**	Addition of templated notes to improve documentation
**Amendment #2/Version date: April 6, 2016**	
Section #/page	Description of change (s)
**5.2.5**	Added FAMCARE, to assess the caregivers' satisfaction with medical care their family member or friend received.
**5.4.2.1**	Added FAMECARE to study calendar
**5.5.1.4**	Added FAMCARE to study procedures
**Amendment #2/Version date: April 22, 2016**	
Section #/page	Description of change (s)
**4.0**	Added Taussig Cancer Center Supportive Oncology Team
**7.1**	Added CCF REDCap
**7.2.3**	Added electronic system used for CCF, and information regarding the Cleveland Clinic PI.
**Continuing Review**	
**5.3.2.1**	Change in wording to make contact optional, based on initial assessment
**3.3**	Caregiver exclusion criteria added to exclude professional, paid caregivers

SCHEMA

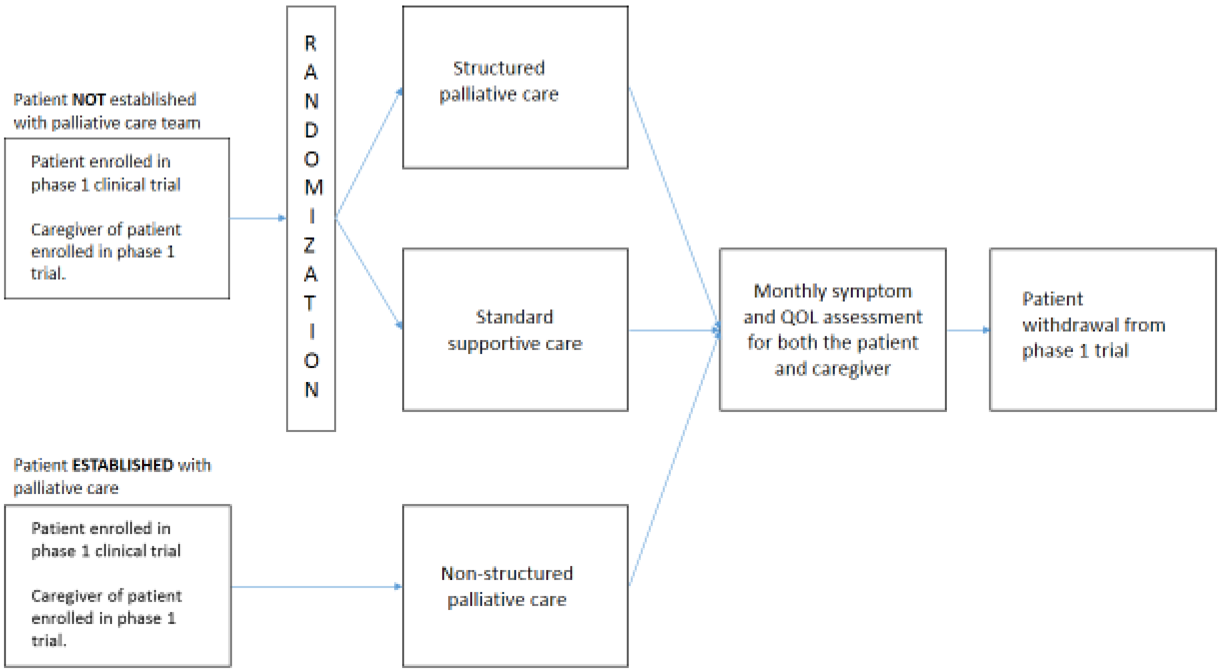

Patient **NOT** established with palliative care team
Patient enrolled in phase 1 clinical trial
Caregiver of patient enrolled in phase 1 trial.
RANDOMIZATION
Structured palliative care
Standard supportive care
Monthly symptom and QOL assessment for both the patient and caregiver
Patient withdrawal from phase 1 trial
Patient **ESTABLISHED** with palliative care
Patient enrolled in phase 1 clinical trial
Caregiver of patient enrolled in phase 1 trial.
Non-structured palliative care

TABLE OF CONTENTS

1.0 INTRODUCTION
1.1 Overview …………………………………………………………………………7
1.2 Background and Rationale…………………………………………………………..7

2.0 OBJECTIVES……………………………………………………………………….11

3.0 RESEARCH SUBJECT SELECTION……………………………………………...11

4.0 RESEARCH SUBJECT ENTRY…………………………………………………...12

5.0 STUDY DESIGN AND METHODS
5.1 Design/Study Type…………………………………………………………………12
5.2 Selection of Instruments……………………………………………………………13
5.3 Description of Intervention ………………………………………………………...14
5.4 Data Collection …………………………………………………………………….16
5.5 Description of Study Process ………………………………………………………18
5.6 Adverse Reactions and Their Management…………………………………...19

6.0 STATISTICAL ANALYSIS ………………………………………………………..21

7.0 RECORDS TO BE KEPT/REGULATORY CONSIDERATIONS ………………...23

8.0 REFERENCES …………………………………………………………………...26

9.0 APPENDICES
Appendix A
Functional Assessment of Cancer Therapy –General (FACT-G)…………………………...29
Appendix B
Memorial Symptom Assessment Scale Short Form (MSAS-SF)…………………………...30
Appendix C
Quality of Life in Life Threatening Illness- Family Carer Version (QOLLTI-F)…………..34
Appendix D
Caregiver Reaction Assessment (CRA)…………………………………………………....36
Appendix E
Note templates for MD/ NP Palliative Care intervention…………………………………...38
Appendix F
Note templates for Psychosocial Intervention………………………………………………40
Appendix G
Randomization Key…………………………………………………………………………42

Appendix H
FAMCARE Scale 44

1.0 INTRODUCTION

1.1 Overview

Cancer patients eligible for early clinical trials are a unique population. These patients have usually exhausted conventional therapeutic options and have limited life expectancy and significant symptom burden, making them appropriate candidates for either early clinical trials or best supportive care. The philosophies of these treatment options are quite different; the goal of Phase 1 clinical trials is to determine the safety, dosing, and side effects of new drugs in humans, while that of palliative care is to improve comfort and quality of life and to provide psychological, emotional, and spiritual support for patients and their families.[1]

While research has shown that the majority of patients enrolled on Phase 1 clinical trials hope to benefit from therapy, these studies are not designed to determine antitumor effectiveness and there is limited expected benefit to patients.[2,3] Although the criteria for enrollment onto Phase 1 studies includes good performance status, these patients have been found to have similar symptom burden to patients not participating in early phase studies.[4,5] In regards to withdrawing patients from early phase trials, the majority of patients come off study because of disease progression or decline in their performance status. Nevertheless, these patients typically have advanced disease and in some circumstances, it may be difficult to discern whether their symptoms or decline in function status are secondary to disease progression or in fact represent treatment toxicity.

We hypothesize that provision of structured palliative care to patients enrolled in Phase 1 clinical trials will decrease their symptom burden, be associated with fewer adverse events, increase duration on trial and improve the quality of life for participants and their family members. Our long term goal is to conduct a prospective randomized Phase III clinical trial of aggressive palliative care versus usual care for patients taking part in Phase 1 trials. As an initial step, we propose a pilot study to obtain preliminary data necessary to develop an appropriate intervention and sample size determination for a subsequent large-scale study.

1.2 Background and rationale

1.2.1 Phase 1 clinical trials: study goals and patient population

Phase 1 clinical trials are the foundation of cancer drug advancement.[6] Their main goal is to evaluate the safety profile of a new agent or new combination and to determine the dose-limiting toxicities and recommended Phase II dose. Patient participation in clinical trials is necessary for new drug development, though current accrual is far from ideal. One of the major barriers to patient participation from a patient perspective is concern for potential side effects.[2,7] One of the hypothesized outcomes of this intervention is to decrease symptom burden and decrease the frequency adverse events, which if true, could potentially be addressed in future studies to aide in patient enrollment.

Eligible patients typically have progressed through standard therapies and have a good Eastern Cooperative Oncology Group (ECOG) performance status (</= 2). Despite their good performance status, participants in Phase 1 clinical trials have similar symptom burden compared

to cancer patients not participating in clinical trials.[4,5] In the study conducted by Finlay et al., the patients enrolled on early phase trial as well as those not enrolled on study, reported a median of five symptoms with equal number and severity of physical and psychological symptoms experienced. When adjusting for the initial ECOG performance status, the study concluded that Phase 1 patients actually experience greater symptom burden then those not on study.[4] A pilot study conducted by Healy et al. described variables which correlate with how well a patient will do on a Phase 1 clinical trial. In their retrospective review, they found that the number of active symptoms (>3) was significantly correlated with increased complications from the experimental treatment.[8] Not surprisingly, the higher number of symptoms resulted in greater decline in functional status as well as increased hospitalizations, both of which are reasons to consider patient withdrawal from the trial.[8] Accordingly, these patients would likely benefit from palliative care services.

While the hope for personal benefit – including remission or even cure - is a major motivation for patients enrolling into Phase 1 clinical trials,[2,3,7,8] the studies are not designed or intended to prolong life or modify the course of the patient's disease. The reported treatment response rates have consistently been less than 5 percent.[9] The life expectancy of patients enrolled in Phase 1 clinical trials is between 5 and 6.5 months.[4] While reasons for patient withdrawal from clinical trials have not been extensively studied, it is assumed that the majority of patients leave a study because of disease progression. However, suffering from undertreated emotional and physical distress likely influences this decision.

In an effort to better characterize the reasons for patient withdrawal from Phase 1 clinical trials and patterns associated with early withdrawal, we performed a medical record review, using our center's clinical trials database (OnCore) of solid tumor oncology patients enrolled onto institutional Phase 1 clinical trials through the Case Comprehensive Cancer Center (CCCC) in the years 2003-2013, excluding data from Cooperative Group or Pharma-sponsored studies, as this data was not included in the OnCore database. Two hundred forty-eight patients were included in our analysis. We included all adverse events regardless of their attribute, as unrelated adverse events are often reasons for study withdrawal. All patients experienced adverse events. The mean number of adverse events experienced was 26.6, with a median of 19 (range: 1-168). Of these adverse events 3023 were potentially addressable, defined as non-laboratory events. One hundred and fifty-one (59.9%) patients discontinued study treatment because of disease progression and 36 (14.3%) because of adverse events. Twenty-two (8.7%) ended participation because they completed the treatment. While this study was limited by the retrospective nature of the data and limitations in clinical documentation, the symptom burden alone confirms a need for palliative care in this population. Unaddressed in the retrospective data as well as many other studies, is the effect of undertreated emotional distress, which in conjunction with undertreated physical symptoms, can confound the assessment of the effects of the experimental and potentially therapeutic agents on quality of life. Addressing factors associated with premature withdrawal, whether by improved management of physical symptoms or increased psychosocial support of patients and families/caregivers, is imperative and may minimize early termination of trials which can impose significant limitations and delays in evaluation of new therapies. Therefore, we propose development of a structured palliative care intervention to address potentially treatable adverse events and improve patient and caregiver quality of life.

1.2.2 Palliative care: Patient and caregiver

The WHO defines palliative care as "an approach that improves the quality of life for patients and their families facing the problems associated with a life-threatening illness, through the prevention and impeccable assessment and treatment of pain and other problems, physical psychosocial and spiritual".[10] Over the last decade, palliative care has been acknowledged as an integral part of providing comprehensive care to patients with cancer.[11] Simultaneously, quality of life has emerged as a key outcome of health related interventions. Through various forms of education, advocacy and policy statements, the oncologic community has made strides to incorporate palliative care into standard clinical practice. The American Society of Clinical Oncology (ASCO) published a position statement in 2009 supporting the vision of incorporating palliative care from the time of diagnosis throughout the experience of cancer.[11] In 2012, ASCO released a consensus clinical opinion that standard oncology care combined with palliative care should be considered early in the course of illness for any patient with metastatic cancer and/or high symptoms burden.[12]

Although the term "palliative care" is frequently used interchangeably with "hospice," there are distinct differences between the two services. Hospice requires that a patient have a terminal diagnosis and has opted to forgo disease-modifying or life-prolonging treatments. While palliative care is commonly provided to patients with terminal diagnoses, it can be used at any point in the disease trajectory and is intended to be used in conjunction with other medical treatments. The perception that hospice and palliative care are one in the same may influence both the physician and patient perception, as well as the offering and acceptance of palliative care services. Several articles have been published regarding Phase 1 patients and whether they have to choose between Phase 1 studies or hospice. Unfortunately, the investigational agents used in Phase 1 trials are considered to be disease directed therapy making these patients, who otherwise would be eligible for hospice, unable to utilize their services. Palliative care advocates have argued that hospice or palliative care should be available to patients enrolled in Phase 1 clinical trials.[4,10,13,14] Nonetheless, due to the complexity of the current reimbursement system under the Center for Medicare and Medicaid Services regulations, access to hospice for this unique group of patients must be forgone. However, no such issue exists to exclude palliative care in the treatment plan.[4,10]

1.2.2.1 The Patient

As the symptom and psychosocial needs of patients with advanced cancer have become increasingly recognized, palliative care programs have been expanded and developed in the cancer care settings.[11,15] Several studies have evaluated palliative care in the outpatient setting, with patients in the intervention arms experiencing improvement in symptoms, mood and overall quality of life. Accordingly, integrated outpatient palliative care has resulted in increased patient satisfaction, improved well-being and decreased symptom burden, including anxiety, depression, fatigue, insomnia, anorexia and nausea.[1,10,11,15,16] In addition to improving patient symptoms and quality of life, the seminal study by Temel et al. demonstrated a 2 month improvement in overall survival in patients with metastatic non-small cell lung cancer who received early palliative care.[1] While the survival improvement is intriguing and has received a lot of attention, the goal of palliative care is neither to extend life nor hasten death.

Patients at the juncture in their disease trajectory when Phase 1 trials are an option, are concurrently at a point when best supportive care or hospice is also an option. There is no clinical reason why disease directed therapy and palliative care cannot be administered concurrently. One study to date has evaluated simultaneous palliative care in patients enrolled on early phase trials. This study demonstrated that the intervention is feasible and did not result in adverse events. While it showed a trend toward improved quality of life, it was not found to be statistically significant, but the study was limited by its size and also lack of randomization. It did demonstrate a wide variability in symptoms over time, signifying a necessity for services that can be adapted to fluctuating patient needs.[17]

1.2.2.2 The Caregiver

The goal of palliative care is to not only optimize the quality of life for persons living with the chronic or terminal illness but also to support and improve the quality of life of their families and caregivers. Throughout the trajectory of each patient's illness, family members or caregivers assume a large part of their care. The caregiver role, particularly when caring for patients in the last months of life, is changing, with the trend toward providing home care to patients with advanced stage cancer.[18] While this trend is consistent with most people's desire to die at home,[10] a great deal of responsibility is placed on the caregiver. Studies have shown that caring for a loved one with a terminal illness at home increases the distress surrounding his or her death.[18]

The caregiving experience is complex, impacting all aspects of the caregiver's life, including physical, emotional and psychological well-being.[19,20] The distress experienced by the friends or family member(s) as caregiver is multidimensional and varies tremendously throughout the patient's disease course. A multitude of factors contribute to the caregiver experience, related not only to changes in the patient condition, but also to how their new role influences the interdependent aspects of daily life. This includes coping with disruptions in the daily routine through providing direct care, performing medical procedures, communicating with healthcare professionals and providing emotional support to the patient as well as family members.[19,20] It also involves restructuring or relinquishing responsibilities related to childcare, employment and financial obligations as well as negotiation of factors related to familial or generational relationships.[20] The extent of burden or impact on quality of life between caregivers varies widely, as it is affected by each individual's baseline coping strategies and support systems.[20]

The adjustment of the caregiver to the role affects not only the caregiver but also the patient and the entire home care support system.[19] Studies have shown that increased symptom burden and decreased level of activity in patients results in more caregiver stress and depression.[19,20] Reciprocally, burden on the family is often one of the patient's main concerns[10] and a patient's level of activity may decrease as his or her caregiver's needs increase.[19] While caregivers of patients enrolled in early clinical trials are not well studied, research has demonstrated that caregivers of patients receiving "aggressive" care are at increased risk of feeling unprepared, becoming depressed and having worse quality of life.[10] One of the goals of this study is to assess the caregiver experience throughout patient enrollment on the Phase 1 study and how simultaneous palliative care influences that experience. We anticipate a positive impact, as

palliative care involvement has been shown to improve caregiver health-related quality of life, particularly in regards to the emotional component.[10]

1.2.3 Rationale for evaluating simultaneous palliative care in patients enrolled on Phase 1 clinical trials

Given the data on the benefits of palliative care, we hypothesize that provision of structured palliative care to patients enrolled in Phase 1 clinical trials will decrease their symptom burden, be associated with fewer adverse events, increase duration on trial and improve the quality of life for participants and their caregivers. Our long term goal is to conduct a prospective randomized Phase III clinical trial of aggressive palliative care versus usual care for patients taking part in Phase 1 trials. As an initial step, we propose a pilot study to obtain preliminary data necessary to develop an appropriate intervention and sample size determination for a subsequent large-scale study.

2.0 OBJECTIVES

2.1 Primary Objective(s): To assess symptoms, adverse events, duration on study, reason for study discontinuation, and quality of life among patients participating in Phase 1 clinical trials who receive structured palliative care, and those who receive standard supportive care. Duration on study will serve as our primary objective for sample size determination.

2.2 Secondary Objective(s):
1. To describe the quantity (e.g. hours), type, and cost of palliative care personnel services used by patients and caregivers receiving structured palliative care.

2. Exploratory:
a) To compare adverse event profiles, duration on study, and quality of life between patients who receive structured palliative care and those receiving standard supportive care.
b) To compare caregiver burden and quality of life between the study arms.

3.0 RESEARCH SUBJECT SELECTION AND ELIGIBILITY

Each of the criteria in the checklist that follows must be met for a patient to be considered eligible for this study. Use the checklist to confirm a patient's eligibility. The checklist must be completed for each patient and must be signed and dated by the treating physician.

3.1 Patient inclusion criteria

Patients must meet all of the following inclusion criteria to be eligible for enrollment

____ 3.1.1 Patients must be enrolled in a Phase 1 clinical trial and be within 2 weeks of starting the experimental therapy or intervention.

____ 3.1.2 Age ≥18 years

____ 3.1.3 Patients are eligible to enroll on this study with or without the enrollment of their caregiver.

3.2 **Patient exclusion criteria:**

The presence of any of the following will exclude a patient from study enrollment
____ 3.2.2 Patients diagnosed with a hematologic malignancy.

3.3 **Caregiver inclusion criteria**

Caregivers must meet all of the following inclusion criteria to be eligible for enrollment
____ 3.3.1 A caregiver who is the person identified by the patient as the one who provides the most regular physical and/or emotional support.

____ 3.3.1 Caregivers must be willing to complete surveys (described below) at baseline and on monthly basis.

3.4 **Caregiver exclusion criteria**

____ 3.4.1 Caregivers who are solely professional, paid caregivers.

3.5 **Inclusion of Women and Minorities**

Both men and women and members of all races and ethnic groups are eligible for this trial.

4.0 RESEARCH SUBJECT ENTRY

All eligible patients who are enrolled into a Phase 1 clinical trial are considered potential participants. These patients will be identified by the Phase 1 team who will notify us of their enrollment.

All subjects consented for this study will be registered in the OnCore Database. For those subjects who are not enrolled, the reason for exclusion must be recorded.

All subjects will be registered through the Case Comprehensive Cancer Center and will be provided a study number by the Study Coordinator.

Once consented, subjects not already established with palliative care will be randomized to receive standard supportive care provided by their treating oncologist or the structured palliative care intervention provided by the Seidman Cancer Center Supportive Oncology Team or by the Taussig Cancer Center Supportive Oncology Team. Randomization will be done using a generated randomization key (see appendix G). The patients already established with palliative care will continue to follow with their providers.

5.0 STUDY DESIGN AND METHODS

5.1 Design/Study Type

A two arm prospective randomized clinical trial of standard supportive care versus structured simultaneous palliative care in patients enrolled in Phase 1 clinical trials. The standard supportive care will be provided by the patients' treating oncologist. The structured palliative care intervention will be provided by the outpatient palliative care team which consists of clinicians (physicians and nurses) trained in palliative care, spiritual care specialists and social workers. Eighty patients will be enrolled and randomized to one of these two groups. Treatment (structured palliative care) will begin after enrollment, either prior to or within two weeks of beginning experimental therapy through the Phase 1 trial, and will continue until the patient leaves the clinical trial. Each patient, and his or her caregiver if enrolled, will complete Symptom and QOL assessment instruments on monthly basis.

There will be a third contemporaneous arm, which will include patients already established with palliative care. These patients will continue to follow with the palliative care team as they have in the past, being seen as clinically indicated. They will not be required to have monthly visits with the palliative care team (as those randomized to the supportive care), but we will collect information on the palliative care resources used including number and frequency of visits, phone calls and services provided. Their caregivers will also be approached, but will receive the standard supportive care services provided by the palliative care team and we will collect information on the services they utilize. Both the patient and caregiver will complete the monthly symptom and QOL assessment instruments.

5.2 Selection of Instruments

5.2.1 Functional Assessment of Cancer Therapy –General (FACT-G)

The FACT-G is a reliable and validated outcome measure, which is widely used to assess health-related quality of life (HRQoL) in clinical research with patients with cancer. It is a 27 item questionnaire with 4 subscales including physical well-being (7 questions), social or family well-being (7 questions), emotional well-being (6 questions) and functional well-being (7 questions). The questions are answered on a five point Likert-type scale ranging from 0 (not at all) to 4 (very much), over a recall period of the 7 days. The responses are summed to create a total score and individual subscale scores with higher scores indicating better HRQOL.[21]

5.2.2 Memorial Symptom Assessment Scale Short Form (MSAS-SF):

The Memorial Symptom Assessment Scale Short Form is extensively used in cancer trials. It is a reliable and valid instrument, developed to assess the common physical and psychological symptoms in patients with cancer. 28 physical symptoms and the frequency of 4 psychologic symptoms during a 7 day time period are evaluated with respect to the distress they cause. The MSAS-SF consists of subscales that describe global symptom distress (GDI), physical symptom distress (PHYS), and psychological distress (PSYCH). The total MSAS-SF score is the average of the 32 symptom scores.[22] The survey takes approximately 5-10 minutes to complete.

5.2.3 Quality of Life in Life Threatening Illness- Family Carer Version (QOLLTI-F)

The QOLLTI-F is a validated and reliable assessment tool, which measures family caregiver quality of life. This tool focuses specifically on caregivers providing care to someone with a life-threatening illness. The questionnaire consists of 16 items covering seven domains including environment, patient condition, quality of care, carer's outlook, carer's own state and financial

worries. A two day time frame is used, as situations for both the patient and caregiver can change rapidly, especially near the end of life. The response scale is an 11 point numerical rating scale which ranges from 0-10, 10 being the best possible situation. The mean score of each domain is then calculated. The questionnaire takes approximately 10 minutes to complete.[18]

There is consensus among QOL researchers that a change of 0.5 standard deviations, usually corresponding to 5-10% of the scale range , represent a minimal clinically important difference.[18] We will use this standard in our study.

By administering the quality of life assessment tools to both the patient and the carer simultaneously, we hope to better understand the relationship between the QOL of the patient and the carer and the impact of the intervention on both.

5.2.4 Caregiver reaction assessment (CRA)

The CRA is a validated and reliable assessment tool used to assess various aspects of caregiver burden.[19,23,24] It is a self-rating scale that consists of 24 items that covers five domains: self-esteem (7 items), family support (5 items), finances (3 items), daily schedule (4 items), and health (4 items). The perceived impact is rated on a 5-point Likert scale, with the format: 1 = strongly disagree, 2 = disagree, 3 = neither agree nor disagree, 4 = agree, 5 = strongly agree. Each subscale is added to a sum score, this is divided by the number of items to reflect an unweighted mean-item score with a range from 1.0 to 5.0. High scores on all subscales indicate negative experience except for self-esteem where a low score indicated a negative experience. The initial CRA did not offer a total score but Grove and colleagues[24] recoded the self-esteem score to allow for a total CRA score to reflect the total caregiver situation. The total CRA score is the sum of the 24 items and higher scores reflect higher caregiver burden.

5.2.5 Family Satisfaction with Advanced Cancer Care (FAMCARE)

FAMCARE is a valid and reliable instrument which has been widely used to assess caregivers satisfaction with the medical care provided to the patient for which they care. It is a unidimiensional model, which covers 4 subdimensions of care including information giving, availability of care, psychological care and physical patient care. It is a 20 item scale which uses a five point Likert scale: very satisfied (1), satisfied (2), undecided (3), dissatisfied (4), and very dissatisfied (5). The questionnaire takes approximately 10 minutes to complete.[25]

5.3 Description of Intervention

The intervention is a providing simultaneous structured palliative care to patients enrolled into Phase 1 clinical trials. Patients randomized to this arm will meet with a specialized palliative care clinician after enrollment into the Phase 1 clinical trial and prior to initiation of the experimental therapy.

5.3.1 Standard Supportive Care (Arm 1)

5.3.1.1 Patient

In the standard supportive care cohort, the supportive care will be provided by the treating oncologist. The frequency of visits and referral to other specialties or services (nutrition, psychiatry, palliative care, etc.) will be at discretion of the treating oncologist. The treating oncologist can refer patients to palliative care. The data collected on patients referred to palliative care will be analyzed, using the intention to treat principle, in the standard supportive care cohort.

5.3.1.2 Caregiver

In the standard supportive care cohort, caregiver support will be provided by the treating oncologist and referral to psychosocial personnel will be at their discretion. The caregivers will be required to complete CRA and QOLLTI-F surveys on a monthly basis, until the patient is off the Phase 1 study.

5.3.2 Structured Palliative Care (Arm 2)

5.3.2.1 Patient

In the structured palliative care cohort, supportive care will be provided by the outpatient palliative care team which includes clinicians with specialized palliative care training, social workers, spiritual care specialists and mental health clinical nurse specialist (CNS). Each patient will meet with a palliative care physician or an advanced practice nurse (APN) at the initial visit which will be within two weeks of starting experimental therapy through the Phase 1 clinical trial. A comprehensive evaluation, including medical, physical and psychosocial assessment will be performed at the initial visit and recommendations made for palliative care treatment, counseling, home support and referral to other specialties. Monthly (every 30 days +/- 7 days) face to face visits with either the physician or APN will be scheduled, until the patient withdrawals from the Phase 1 clinical trial. At each in person visit, the results from the MSAS-SF will be used to help the team determine which symptoms need to be addressed. After their initial visit, the patient may be contacted the following week by phone to assess for symptoms or questions, based on needs identified at the time of their initial assessment. Patients will have open communication with their palliative care providers and will be able to communicate with their provider by phone or be seen on a more frequent basis if necessary. As at the initial visit, at each follow up visit, the palliative care team will address and make recommendations for symptoms. Treatment decisions or suggestions will be communicated to the primary oncologist and Phase 1 clinical trial team through email on the day of the visit. These decisions will also be documented in the medical record using a follow up note template (see appendix). The palliative care provider will also periodically address goals of care and end of life planning. Advanced care planning discussions will be summarized and DNAR orders documented in the follow up note.

5.3.2.2 Caregiver

In the structured simultaneous palliative care cohort, the caregiver will communicate (either in person or by telephone) with a psychosocial provider (social worker, mental health CNS, spiritual care provider) and an in person visit scheduled, within 2-3 weeks of patient enrollment onto the Phase 1 clinical trial. At the initial visit, the caregiver will complete the CRA and QOLLTI-F questionnaires. The caregiver will be required to complete the aforementioned assessment surveys monthly, until the patient comes off of the Phase 1 clinical trial. The caregiver will then have the option to communicate with the supportive oncology team via

telephone or in person visits, depending on their needs and preference. Summaries of these visits will be documented in the medical record using a template note (see appendix).

5.3.3 Patients established with palliative care prior to enrollment on phase 1 study (Arm 3)
5.3.3.1 Patient
Patients established with the palliative care team prior to enrollment on the phase 1 clinical trial will continue to see both their treating oncologist and palliative care provider as they have in past. There will not be scheduled visits as in the structured simultaneous care cohort. Patients will be seen at the discretion of the palliative care provider as clinically indicated and referral to other specialties or services will be at their discretion. As in the structured simultaneous care cohort, treatment decisions or suggestions will be communicated to the primary oncologist and the phase 1 clinical trial team through email on the day of visit. The visits will be documented in the medical record using the follow up note template (see appendix). The patients will be required to complete FACT-G and MSAS-SF surveys on a monthly basis until they are off the phase 1 study.

5.3.3.2 Caregiver
The caregivers in this arm will be provided the standard supportive care services offered by the palliative care team and the treating oncologist. There will not be scheduled visits or discussions as in the structured simultaneous care cohort. Meeting and communication with these caregivers will be documented in the medical record using a template note (see appendix). The caregivers will be required to complete CRA and QOLLTI-F surveys on a monthly basis, until the patient is off the Phase 1 study.

5.4 Data Collection

5.4.1 Screening evaluation

A screening evaluation will be used to determine the eligibility of each subject for study inclusion. Those who have established care with a palliative care physician or have hematologic malignancy will be excluded.

For the patient, the following will be obtained at baseline from the medical record:

- Demographics
- Medical history including site of cancer, stage of disease and lines of prior therapy.

For the patient, the following will be obtained/performed at their initial visit

- Physical exam including height and weight
- Medication use, this includes all prescription medications, over the counter medications and herbal supplements
- ECOG performance status
- Reason for enrollment into the early phase clinical trial
- Baseline quality of life assessment through the use of FACT-G
- Baseline symptom assessment through the use of MSAS

For the caregiver, the following should be obtained at baseline

- Caregiver relationship to patient
- Sociodemographic information including: age, sex, education, occupation, employment status, income.
- Amount of hours spent with patient per week
- Measurement of caregiver quality of life through the use of the QOLLTI-F
- Measurement of caregiver burden through the use of the CRA

5.4.2 Treatment Period

Treatment (simultaneous palliative care verses standard supportive care) will begin following enrollment and will continue until the patient either completes or withdrawals from the Phase 1 clinical trial.

Patients in the intervention arm will have monthly scheduled visits with a palliative care clinician in addition to their appointments with their treating oncologist. Patients will have open communication with the palliative care clinician and can be seen more frequently if necessary. Referral to other specialties (psychology, psychiatry, nutrition, etc.) will be at the discretion of the palliative care clinician. The frequency of additional visits as well as referrals will be documented in the medical record.

All patients enrolled in the trial will complete the assessment instruments including the FACT-G and MSAS on a monthly basis.

All caregivers enrolled in the trial will complete the assessment instruments including the QOLLTI-F and CRA on a monthly basis.

Palliative care resources: Members of the palliative care team will record their hours spent with the patient and his/her caregiver weekly.

If one of the participants (patient/caregiver) misses an appointment, this will be documented and they will be contacted to schedule an appointment and/or time to complete the assessment tool as soon as possible.

5.4.2.1 Calendar for study participants
Calendar for patients

Study Week	Screen	Monthly palliative care visits	Additional visits for uncontrolled or new symptoms or issues	Withdrawal from trial
Required assessments				
Informed consent	X			
Demographics	X			

Medical history and physical exam[1]	X			
ECOG PS	X	X	X	X
Reason for enrollment	X			
Symptom assessment	X	X	X	X
Height[2], weight	X	X	X	
MSAS*	X	X		X
FACT-G	X	X		X
Reason for withdrawal				X

[1]Including medication history
[2]Height need only be measured at screening
*The MSAS will be administered prior to the FACT-G

Calendar for caregivers

Study Week	Baseline quality of life	Monthly patient visits	At time of patient withdrawal from trial	After the patient is off study
Required assessments				
CRA*	X	X	X	
QOLLTI-F	X	X	X	
Famcare				X
Number of hours spent with patient per week	X	X	X	

*****The CRA will be administered prior to the QOLLTI-F

The intervention will end once the patient completes or withdrawals from the phase 1 clinical trial, enrolls in hospice or dies. For descriptive purposes, for each patient, we will collect data on hospice enrollment, place and time of death and whether treatment was received in last 30 days of life. We plan to obtain this information if possible through the medical record and other publically available information including obituaries and the national death registry. If we are not able to get this information through these records we will attempt to contact the patients treating oncologist or primary care physician and if still unable to get the information will call the patient/caregiver periodically to assess the patient’s status.

5.5 Description of Study Process

<u>5.5.1 Instrument Administration:</u> The following assessment tools will be administered by a research assistant or other member of the research team.

5.5.1.1 Memorial Symptom Assessment scale short form (MSAS-SF).
Change in symptom distress will be assessed through the MSAS-SF at baseline, followed by monthly assessments until the patient comes off study. Estimated time to complete is approximately 5-10 minutes.

5.5.1.2 FACT-G
QOL will be assessed through the use of the FACT-G at baseline, followed by monthly visits until the patient comes off study. Estimated time to complete the assessment tool is approximately 15-20 minutes.

For the patient the MSAS will be administered before the FACT-G in an attempt to get a more accurate assessment of quality of life. Identification and reporting of symptoms may prompt patients to reflect on how their symptoms are impacting their quality of life.

5.5.1.3 CRA and QOLLTI-F:
Caregiver burden and quality of life will be assessed through the use of the CRA and QOLLTI-F. These will be administered on a monthly basis. The QOLLTI-F takes approximately 10 minutes to complete and the CRA takes less than 10 minutes to complete.

For the caregiver the CRA will be administered before the QOLLTI-F in an attempt to get a more accurate assessment of quality of life. Identification of factors that contribute to caregiver burden may prompt caregivers to reflect on how their symptoms are impacting their quality of life.

The rationale for serial assessments is to provide a more accurate representation of change in symptom burden and symptom interrelationship.[26]

5.5.1.4 FAMCARE
The caregiver satisfaction with the care their friend or family member received will be assessed through the use of the FAMCARE. This will be administered once the patient has come off of the study and takes approximately 10 minutes to complete.

<u>5.5.2 Intervention Administration</u>
5.5.2.1 The participants randomized to the structured palliative care arm will meet with either the palliative care physician or the RN at the time of enrollment. Each participant will then have scheduled visits with either the palliative care physician or RN every month until he or she withdrawals from the Phase 1 trial. Participants in the structured palliative care arm will have open communication with their palliative care provider to address symptoms which are not controlled or new symptoms and additional visits can be scheduled if necessary. Referral to other specialties will be at the discretion of the palliative care clinician and will be documented and analyzed.

5.5.2.2 The caregivers randomized to the structured palliative care arm will meet with a member of the palliative care team within 2-3 weeks of the patient's enrollment onto the phase 1 clinical trial. They will then have the option to communicate with the supportive oncology team in person on the day of the patient's visit, or on a separate day, whichever is convenient for them. They can also communicate with supportive oncology team by phone if preferred. This

communication will be at least monthly but can be more frequent if necessary. The counseling and support services necessary will be documented.

5.5.3 Special Concerns

Discussions with the palliative care physician regarding goals of care and end of life decisions may cause emotional distress such as anxiety or depression.

5.5.4 Compensation

Study subjects will not be compensated for their participation in this study. Patients receiving structured palliative care intervention will not be billed for the visits with the palliative care team. Any medications prescribed will be billed through their insurance.

5.6 Adverse Reactions and Their Management:

An **adverse event** (AE) is any unfavorable or unintended event, physical or psychological, associated with a research study, which causes harm or injury to a research participant as a result of the participant's involvement in a research study. The event can include abnormal laboratory findings, symptoms, or disease associated with the research study. The event does not necessarily have to have a causal relationship with the research, any risk associated with the research, the research intervention, or the research assessments.

Adverse events may be the result of the interventions and interactions used in the research; the collection of identifiable private information in the research; an underlying disease, disorder, or condition of the subject; and/or other circumstances unrelated to the research or any underlying disease, disorder, or condition of the subject.

External adverse events are adverse events experienced by subjects enrolled in multicenter clinical trials at sites other than the site(s) over which the Institutional Review Board has jurisdiction.

Internal adverse events are adverse events experienced by subjects enrolled at the site(s) under the IRB's jurisdiction for either multicenter or single-center research projects.

5.6.1 Reporting Adverse Events

All participating investigators will assess the occurrence of AEs throughout the subject's participation in the study. The clinical course of each event will be followed until resolution, stabilization, or until it has been determined that the palliative care intervention is not the cause. Adverse events will be reported to the IRB according to institutional policy. This study is being done in conjunction with various Phase 1 studies many of the adverse events will be attributable to the experimental agent. As part of our study, we will record all adverse events and will attribute the adverse events appropriately. Accordingly, a great majority will likely not be attributable to our study intervention.

The investigator is responsible for ensuring that all adverse events observed by the investigator or reported by the subject which occur after the subject has signed the informed consent are fully recorded in the subject's case report form, subject's medical records, and/or any other institutional requirement. Source documentation must be available to support all adverse events.

The investigator will provide the following for all adverse events:

- Description of the event
- Date of onset, duration and resolution
- Attribution of relatedness to the palliative care intervention
- Action taken as a result of the event
- Outcome of event

5.6.2 Anticipated Reactions

Medical risk of study participation are expected to be extremely low. Patients may experience emotional distress when contemplating or discussing end of life issues. We do not anticipate any physical adverse events from participating in this study.

5.6.3 Reaction Management

Patients will meet with members of palliative care team regularly and will have open communication with that team. Education and counseling will be provided during times of emotional distress to help patients cope with their emotions.

6.0 **STATISTICAL ANALYSIS**

This is a pilot study to generate preliminary, descriptive data. As such the primary and secondary endpoints will be descriptive.

6.1 Definition of primary objective(s)/endpoint(s):

6.1.1 Measures of central tendency for adverse events, days on study and reason for study discontinuation for patients (both parent study reported and patient reported) who received structured palliative care, non-structured supportive care and those who received standard supportive care. The categorical data such as symptoms, AE, reason for study discontinuation, type of personnel services (objectives 1 and 2) will be tabulated for each study arm; and the continuous data such as duration on study and QOL, etc., summarized by mean and standard deviation. We will perform statistical inferences/analysis to examine patterns and associations among variables and our primary outcome (duration on study). The association of the duration on study with continuous measurements, such as age, number of co-morbidities will be estimated using Spearman correlation coefficient in the initial univariate analysis. The important covariates identified in the univariate analysis will be further evaluated using multivariable regression analysis where multiple factors are considered simultaneously. Logistic regression will identify predictors of adverse events. We will pay special attention to reason for off study and its association with duration on study. As a secondary step, we will further categorize AE as modifiable (primarily symptom experience) and non-modifiable (laboratory abnormalities). The data will be re-analyzed to identify predictors of each type of adverse event and influence on study duration, particularly estimating the effect of modifiable adverse events on study duration, accounting for other confounders using a regression model.

An exploratory, weighted adverse event score will be calculated for each patient. This score will be based on the number of adverse events as well as the adverse event grade; adverse event score

$= \sum (N_{AE} * G_{AE})$. N denotes the adverse event number, G denotes grade, AE denotes adverse event. This is an exploratory analytic approach, attempting to represent the adverse events experienced by a patient. While it may not take into account the differences that exist between different adverse events of the same grade, or the clinical significance of the non-linear difference between grades, it offers a single measurement to characterize the total symptom experience of the patient and may correlate with clinical trial outcomes including HRQOL.

6.1.2 The Fact-G will be used to measure patient quality of life. Quality of life (total and subscale scores) will be measured at baseline and monthly thereafter. Trends and change scores will be used to examine temporal patterns. For all analyses, demographic and clinical characteristics (age, cancer type, type of therapy, achieving dose-limiting toxicity) will be examined as covariates.

6.1.3 The MSAS will be used to assess patient symptom burden. As with the FACT-G, symptom burden will be measured at baseline and monthly thereafter. Trends and change scores will be used to examine temporal patterns. For all analyses, demographic and clinical characteristics (age, cancer type, type of therapy, achieving dose-limiting toxicity) will be examined as covariates.

6.2 Definition of secondary outcome(s)/endpoint(s):

6.2.1 Mean number of hours, type of palliative care services utilized by patients and caregivers who received structured palliative care (arm 2) compared to those who received standard supportive care (arm 1) and those who were already established with palliative care and received non-structured palliative care (arm 3). Again this analysis will be descriptive, reporting measures of central tendency

6.2.2 Exploratory endpoints:

a) Frequency of adverse events of patients receiving structured supportive care, non-structured supportive care and usual supportive care
b) Days on study
c) Change in patient symptom burden and quality of life as assessed through the FACT-G and MSAS score when compared to baseline between patients who received structured palliative care and those who received standard supportive care.
d) Change in caregiver burden and quality of life as assessed through change in CRA and QOLLTI-F score when compared to baseline between those who received structured palliative care and those received standard supportive care.

6.3 Sample size justification:

Based on Phase 1 clinical trial enrollment at the study site over the past two years, we project approximately 100 patients to be available for enrollment. In the team's previous studies with advanced cancer patients, we demonstrated an average refusal rate of 30%.[24] Because all potential subjects will have indicated interest in participating in clinical research (the Phase 1 trial), we

expect our refusal rate to be somewhat lower, at 20%. This will yield a projected enrollment of 80 patients/year, sufficient for meeting our primary descriptive objective. Again, based on our previous trial of a supportive care team for patients who were not participating in Phase 1 trials, we expect withdrawal from the research procedures of this study (palliative care consultation and questionnaire completion) to be less than 5%. In terms of our exploratory objective, based on the retrospective data on patients under standard supportive care, the average time on trial is 92.7 day (SD: 103.1). We expect the structured palliative care arm will increase the duration on trial by at least 30 days. By randomized Phase II screening design [2], a two group one-sided T-test with a 0.2 significance level will have 80% power to detect the difference of 40 days (effect size = 0.388) on trial between two arms when the sample size in each treatment arm is 38.

7.0 RECORDS TO BE KEPT / REGULATORY CONSIDERATIONS

7.1 Data Reporting

The OnCore Database will be utilized, as required by the Case Comprehensive Cancer Center, to provide data collection for both accrual entry and trial data management. OnCore is a Clinical Trials Management System housed on secure servers maintained at Case Western Reserve University. OnCore properly used is compliant with Title 21 CFR Part 11. Access to data through OnCore is restricted by user accounts and assigned roles. Once logged into the OnCore system with a user ID and password, OnCore defines roles for each user which limits access to appropriate data. User information and password can be obtained by contacting the OnCore Administrator at OnCore-registration@case.edu.

OnCore is designed with the capability for study setup, activation, tracking, reporting, data monitoring and review, and eligibility verification. This study will utilize electronic Case Report Form completion in the OnCore database. A calendar of events and required forms are available in OnCore.

UH REDCap and CCF REDCap will also be used to manage patient data. This is password protected. Only study staff will have access to these databases.

7.2 Regulatory Considerations

The study will be conducted in compliance with ICH guidelines and with all applicable federal (including 21 CFR parts 56 & 50), state or local laws.

7.2.1 Written Informed consent

Provision of written informed consent must be obtained prior to any study-related procedures.
Since we plan to obtain assessment measures in patients and their caregivers, two consent forms will be developed. The Principal Investigator will ensure that the subject and the subjects' caregiver are given full and adequate oral and written information about the nature, purpose, possible risks and benefits of the study. The principal investigator will also ensure that both the subject and his or her caregiver are aware of their financial responsibility. Subjects must also be

notified that they are free to discontinue from the study at any time. The subject should be given the opportunity to ask questions and allowed time to consider the information provided.

The original, signed written Informed Consent Form for both the patient and his or her caregiver must be kept with the Research Chart in conformance with the institution's standard operating procedures. A copy of the signed written Informed Consent Form must be given to the subject.

7.2.2 Subject Data Protection

In accordance with the Health Information Portability and Accountability Act (HIPAA), a subject must sign an authorization to release medical information to the sponsor and/or allow the sponsor, a regulatory authority, or Institutional Review Board access to subject's medical information that includes all hospital records relevant to the study, including subjects' medical history.

7.2.3 Accessing Electronic Medical Records for University Hospitals Health System

This study will access electronic medical records systems to obtain medical information for the subjects enrolled to this study.

In order to insure patient safety, investigators and study personnel must have up-to-the-minute health information for subjects enrolled to this study. Therefore, electronic medical records must be utilized to obtain medical information in a timely manner.

At University Hospitals the following electronic systems will be used: The Athena program to access scheduling information; UH Physician Portal to access lab results and physician notes; PACS to access radiological imaging results; and MySecureCare (Sunrise Clinical Manager) to access some or all of the above information when this application is fully functional.

At the Cleveland Clinic the EPIC electronic system will be used.
Access to these systems is required for the life of this research study.

Information obtained from electronic systems will be copied into the Seidman Cancer Center and Taussig Cancer Center Clinical Trials Unit research chart and/or printed (lab results, physician notes, etc.) and stored in the research chart. Research charts are kept secure and destroyed according to UH and CCF policy. All parties involved will abide by all local laws and regulations.

Study data will be obtained by the study PIs, co-investigators, study coordinator, and/or data manager for this study via password-protected login. The PI, Michelle Treasure, MD is a Case Western Reserve University employee with a University Hospitals email address and IT&S log on ID and Password. For the Cleveland Clinic, the PI, Dale Shepard, MD is Cleveland Clinic employee with a Cleveland Clinic email address and IT&S log on ID and password. All study personnel involved in this research will adhere to the UH/CCF policies regarding confidentiality and Protected Health Information.

7.2.4 Retention of records

The Principal Investigators of The Case Comprehensive Cancer Center supervise the retention of all documentation of adverse events, case report forms, source documents, records of study drug receipt and dispensation, and all IRB correspondence for as long as needed to comply with national and international regulations and the institution in which the study will be conducted, or for the period specified by the sponsor, whichever is longer. No records will be destroyed until the Principal Investigator confirms destruction is permitted.

7.2.5 Audits and inspections

Authorized representatives of the sponsor, a regulatory authority, an Independent Ethics Committee (IEC) or an Institutional Review Board (IRB) may visit the center to perform audits or inspections, including source data verification. The purpose of an audit or inspection is to systematically and independently examine all study-related activities and documents to determine whether these activities were conducted, and data were recorded, analysed, and accurately reported according to the protocol, Good Clinical Practice (GCP), guidelines of the International Conference on Harmonization (ICH), and any applicable regulatory requirements.

7.2.6 Data Safety and Monitoring Plan

This protocol will adhere to the policies of the Case Comprehensive Cancer Center Data and Safety Monitoring Plan in accordance with NCI regulations.

8.0 REFERENCES
Bibliography

1. Temel JS, Greer JA, Muzikansky A, et al. Early palliative care for patients with metastatic non-small-cell lung cancer. *N. Engl. J. Med.* 2010;363(8):733–42. Available at: http://www.ncbi.nlm.nih.gov/pubmed/20818875. Accessed September 28, 2013.

2. Van der Biessen DAJ, Cranendonk MA, Schiavon G, et al. Evaluation of patient enrollment in oncology phase I clinical trials. *Oncologist*. 2013;18(3):323–9. Available at: http://www.ncbi.nlm.nih.gov/pubmed/23429738. Accessed October 3, 2013.

3. Sulmasy DP, Astrow AB, He MK, et al. The culture of faith and hope: patients' justifications for their high estimations of expected therapeutic benefit when enrolling in early phase oncology trials. *Cancer*. 2010;116(15):3702–11. Available at: http://www.pubmedcentral.nih.gov/articlerender.fcgi?artid=3644988&tool=pmcentrez&rendertype=abstract. Accessed October 3, 2013.

4. Finlay E, Lu HL, Henderson H, O'Dwyer PJ, Casarett DJ. Do phase 1 patients have greater needs for palliative care compared with other cancer patients? *Cancer*. 2009;115(2):446–53. Available at: http://www.ncbi.nlm.nih.gov/pubmed/19107769. Accessed September 28, 2013.

5. Hui D, Parsons H, Nguyen L, et al. Timing of palliative care referral and symptom burden in phase 1 cancer patients: a retrospective cohort study. *Cancer*. 2010;116(18):4402–9. Available at: http://www.pubmedcentral.nih.gov/articlerender.fcgi?artid=2936661&tool=pmcentrez&rendertype=abstract. Accessed September 28, 2013.

6. Dowlati A, Kundranda M, Manda S, Patrick L, Fu P. Temporal evolution of patient characteristics enrolled on phase I trials. *Invest New Drugs*. 2011;29(2):312–315. Available at: http://www.ncbi.nlm.nih.gov/pubmed/19997961.

7. Meropol NJ, Buzaglo JS, Millard J, et al. Barriers to clinical trial participation as perceived by oncologists and patients. *J. Natl. Compr. Canc. Netw.* 2007;5(8):655–64. Available at: http://www.ncbi.nlm.nih.gov/pubmed/17927923. Accessed October 3, 2013.

8. Healy JM, Patel T, Lee S, Sanchez-Reilly S. Do symptoms matter when considering patients for phase I clinical trials?: a pilot study of older adults with advanced cancer. *Am. J. Hosp. Palliat. Care*. 2011;28(7):463–6. Available at: http://www.ncbi.nlm.nih.gov/pubmed/21398266. Accessed October 3, 2013.

9. Cheng JD, Hitt J, Koczwara B, et al. Impact of quality of life on patient expectations regarding phase I clinical trials. *J Clin Oncol*. 2000;18(2):421–428. Available at: http://www.ncbi.nlm.nih.gov/pubmed/10637258.

10. Rocque GB, Cleary JF. Palliative care reduces morbidity and mortality in cancer. *Nat. Rev. Clin. Oncol.* 2013;10(2):80–9. Available at: http://www.ncbi.nlm.nih.gov/pubmed/23247373. Accessed September 28, 2013.

11. Ferris FD, Bruera E, Cherny N, et al. Palliative cancer care a decade later: accomplishments, the need, next steps -- from the American Society of Clinical Oncology. *J. Clin. Oncol.* 2009;27(18):3052–8. Available at: http://www.ncbi.nlm.nih.gov/pubmed/19451437. Accessed September 18, 2013.

12. Smith TJ, Temin S, Alesi ER, et al. American Society of Clinical Oncology provisional clinical opinion: the integration of palliative care into standard oncology care. *J. Clin. Oncol.* 2012;30(8):880–7. Available at: http://www.ncbi.nlm.nih.gov/pubmed/22312101. Accessed September 28, 2013.

13. Byock I, Miles SH. Hospice benefits and phase I cancer trials. *Ann. Intern. Med.* 2003;138(4):335–7. Available at: http://www.ncbi.nlm.nih.gov/pubmed/12585832. Accessed October 3, 2013.

14. Casarett DJ, Karlawish JHT, Henry MI, Hirschman KB. Must patients with advanced cancer choose between a Phase I trial and hospice? *Cancer*. 2002;95(7):1601–4. Available at: http://www.ncbi.nlm.nih.gov/pubmed/12237931. Accessed October 7, 2013.

15. Follwell M, Burman D, Le LW, et al. Phase II study of an outpatient palliative care intervention in patients with metastatic cancer. *J. Clin. Oncol.* 2009;27(2):206–13. Available at: http://www.ncbi.nlm.nih.gov/pubmed/19064979. Accessed September 28, 2013.

16. Bakitas M, Lyons KD, Hegel MT, et al. Effects of a palliative care intervention on clinical outcomes in patients with advanced cancer: the Project ENABLE II randomized controlled trial. *JAMA*. 2009;302(7):741–9. Available at: http://www.pubmedcentral.nih.gov/articlerender.fcgi?artid=3657724&tool=pmcentrez&rendertype=abstract. Accessed September 29, 2013.

17. Meyers FJ, Linder J, Beckett L, Christensen S, Blais J, Gandara DR. Simultaneous care: a model approach to the perceived conflict between investigational therapy and palliative care. *J. Pain Symptom Manage.* 2004;28(6):548–56. Available at: http://www.ncbi.nlm.nih.gov/pubmed/15589080. Accessed September 23, 2013.

18. Cohen R, Leis AM, Kuhl D, Charbonneau C, Ritvo P, Ashbury FD. QOLLTI-F: measuring family carer quality of life. *Palliat Med*. 2006;20(8):755–767. Available at: http://www.ncbi.nlm.nih.gov/pubmed/17148530.

19. Deeken JF, Taylor KL, Mangan P, Yabroff KR, Ingham JM. Care for the caregivers: a review of self-report instruments developed to measure the burden, needs, and quality of life of informal caregivers. *J Pain Symptom Manag.* 2003;26(4):922–953. Available at: http://www.ncbi.nlm.nih.gov/pubmed/14527761.

20. Given B, Wyatt G, Given C, et al. Burden and depression among caregivers of patients with cancer at the end of life. *Oncol. Nurs. Forum*. 2004;31(6):1105–17. Available at: http://www.pubmedcentral.nih.gov/articlerender.fcgi?artid=1315286&tool=pmcentrez&rendertype=abstract. Accessed October 6, 2013.

21. Yanez B, Pearman T, Lis CG, Beaumont JL, Cella D. The FACT-G7: a rapid version of the functional assessment of cancer therapy-general (FACT-G) for monitoring symptoms and concerns in oncology practice and research. *Ann. Oncol.* 2013;24(4):1073–8. Available at: http://www.ncbi.nlm.nih.gov/pubmed/23136235. Accessed October 7, 2013.

22. Portenoy RK, Thaler HT, Kornblith AB, et al. The Memorial Symptom Assessment Scale: an instrument for the evaluation of symptom prevalence, characteristics and distress. *Eur J Cancer*. 1994;30A(9):1326–1336. Available at: http://www.ncbi.nlm.nih.gov/pubmed/7999421.

23. Barnes CL, Given BA, Given CW. Caregivers of elderly relatives: spouses and adult children. *Heal. Soc Work*. 1992;17(4):282–289. Available at: http://www.ncbi.nlm.nih.gov/pubmed/1478554.

24. Grov EK, Fossa SD, Tonnessen A, Dahl AA. The caregiver reaction assessment: psychometrics, and temporal stability in primary caregivers of Norwegian cancer patients in late palliative phase. *Psychooncology*. 2006;15(6):517–527. Available at: http://www.ncbi.nlm.nih.gov/pubmed/16189843.

25. Kristjanson LJ. Validity and reliability testing of the FAMCARE scale: measuring family satisfaction with advanced cancer care. Soc Sci Med 1993;36(5):693–701.

26. Kirkova J, Davis MP, Walsh D, et al. Cancer symptom assessment instruments: a systematic review. *J Clin Oncol*. 2006;24(9):1459–1473. Available at: http://www.ncbi.nlm.nih.gov/pubmed/16549841.

9.0 APPENDICES

Appendix A

FACT-G (version 4)

Below is a list of statements that other people with your illness have said are important**. Please circle or mark one number per line to indicate your response as it applies to the past 7 days.**

	**PHYSICAL WELL-BEING**	**Not at all**	**A little bit**	**Som e- what**	**Quite a bit**	**Very much**
GP1	I have a lack of energy	0	1	2	3	4
GP2	I have nausea	0	1	2	3	4
GP3	Because of my physical condition, I have trouble meeting the needs of my family	0	1	2	3	4
GP4	I have pain	0	1	2	3	4
GP5	I am bothered by side effects of treatment	0	1	2	3	4
GP6	I feel ill	0	1	2	3	4
GP7	I am forced to spend time in bed	0	1	2	3	4

	**SOCIAL/FAMILY WELL-BEING**	**Not at all**	**A little bit**	**Som e- what**	**Quite a bit**	**Very much**
GS1	I feel close to my friends	0	1	2	3	4
GS2	I get emotional support from my family	0	1	2	3	4
GS3	I get support from my friends	0	1	2	3	4
GS4	My family has accepted my illness	0	1	2	3	4
GS5	I am satisfied with family communication about my illness	0	1	2	3	4
GS6	I feel close to my partner (or the person who is my main support)	0	1	2	3	4

Q1	*Regardless of your current level of sexual activity, please answer the following question. If you prefer not to answer it, please mark this box ☐ and go to the next section*					
GS7	I am satisfied with my sex life	0	1	2	3	4

Please circle or mark one number per line to indicate your response as it applies to the past 7 days.

	EMOTIONAL WELL-BEING	Not at all	A little bit	Some-what	Quite a bit	Very much
GE1	I feel sad..	0	1	2	3	4
GE2	I am satisfied with how I am coping with my illness....	0	1	2	3	4
GE3	I am losing hope in the fight against my illness	0	1	2	3	4
GE4	I feel nervous...	0	1	2	3	4
GE5	I worry about dying..	0	1	2	3	4
GE6	I worry that my condition will get worse.....................	0	1	2	3	4

Appendix B

MEMORIAL SYMPTOM ASSESSMENT SCALE – Short Form [MSAS-SF]

I. INSTRUCTIONS: Below is a list of symptoms. If you had the symptom DURING THE PAST WEEK, please check Yes. If you did have the symptom, please check the box that tells us how much the symptom DISTRESSED or BOTHERED you.

Check *all* the symptoms you have had during the PAST WEEK.	Yes [✓]	➔➔ IF YES: How much did it DISTRESS or BOTHER you? Not at All [0]	A little Bit [1]	Some-what [2]	Quite a Bit [3]	Very Much [4]
Difficulty concentrating						
Pain						
Lack of energy						
Cough						
Changes in skin						
Dry mouth						
Nausea						
Feeling drowsy						
Numbness/tingling in hands and feet						
Difficulty sleeping						
Feeling bloated						
Problems with urination						
Vomiting						
Shortness of breath						
Diarrhea						
Sweats						
Mouth sores						
Problems with sexual interest or activity						
Itching						
Lack of appetite						
Dizziness						
Difficulty swallowing						
Change in the way food tastes						

MEMORIAL SYMPTOM ASSESSMENT SCALE – Short Form [MSAS-SF]

I. INSTRUCTIONS: Below is a list of symptoms. If you had the symptom DURING THE PAST WEEK, please check Yes. If you did have the symptom, please check the box that tells us how much the symptom DISTRESSED or BOTHERED you.

		➔➔ IF YES: How much did it DISTRESS or BOTHER you?				
**Check *all* the symptoms you have had during the PAST WEEK.**	**Yes [✓]**	**Not at All [0]**	**A little Bit [1]**	**Some-what [2]**	**Quite a Bit [3]**	**Very Much [4]**
Hair loss						
Constipation						
Swelling of arms or legs						
"I don't look like myself"						
**If you had any other symptoms during the PAST WEEK, please list them below, and indicate how much the symptom DISTRESSED or BOTHERED you.** 1. ____________						
2.____________						

II. Below are other commonly listed symptoms. Please indicate if you have had the symptom DURING THE PAST WEEK, and if so, how OFTEN it occurred.

		➔➔ IF YES, How OFTEN did it occur?			
**Check *all* the symptoms you have had during the PAST WEEK**	**Yes [✓]**	**Rarely [1]**	**Occasionally [2]**	**Frequently [3]**	**Almost Constantly [4]**
Feeling sad					
Worrying					
Feeling irritable					
Feeling nervous					

Appendix C

QOLLTI-F©: Quality of Life During Serious Illness – Family Carers

STUDY IDENTIFICATION #: __________ DATE: __________

Day/Month/Year

PLEASE READ THESE INSTRUCTIONS BEFORE ANSWERING THE QUESTIONNAIRE

There are no right or wrong answers. Honest answers will be most helpful.
This questionnaire includes a series of statements that we would like you to respond to by choosing a number from 0 to 10.
These numbers extend from one extreme answer (for example, 'not at all') to its opposite (for example, 'completely').
Please choose or circle the number between 0 and 10 that best represents how you feel.

Note that sometimes the best situation is at the 0 end of the scale, and sometimes the best situation is at the 10 end of the scale.

We are interested in learning about your OVERALL quality of life, so please consider any issues that affect you, even if they are not related to your caregiving role.

A blank in a sentence refers to the person you are caring for, but please do not write any name to keep this confidential.

1. Over the past two days (48 hours) I wondered if the place _____ was staying (home, hospital, other) was the right place to be:

never 0 1 2 3 4 5 6 7 8 9 10 always

2. Over the past two days (48 hours) I had the privacy I wanted:

not at all 0 1 2 3 4 5 6 7 8 9 10 completely

3. Over the past two days (48 hours) the condition of _____ was distressing to me:

not often 0 1 2 3 4 5 6 7 8 9 10 always

4. Over the past two days (48 hours) the amount of control I had over my life was:

not a problem 0 1 2 3 4 5 6 7 8 9 10 a huge problem

5. Over the past two days (48 hours) I had time to take care of myself:

never 0 1 2 3 4 5 6 7 8 9 10 always

6. Over the past two days (48 hours) I was able to think clearly:

not often 0 1 2 3 4 5 6 7 8 9 10 always

7. Over the past two days (48 hours) physically I felt:

extremely poor 0 1 2 3 4 5 6 7 8 9 10 extremely good

8. Over the past two days (48 hours) emotionally I felt:

extremely poor 0 1 2 3 4 5 6 7 8 9 10 extremely good

9. Over the past two days (48 hours) being able to provide care or company for____ made me feel good:

rarely or never 0 1 2 3 4 5 6 7 8 9 10 always

10. Over the past two days (48 hours) I was comforted by my outlook on life, faith, or spirituality:

not at all 0 1 2 3 4 5 6 7 8 9 10 completely

11. Presently I feel that my life has meaning:

very little meaning 0 1 2 3 4 5 6 7 8 9 10 very much meaning

For questions 12 and 13, if you did not make important decisions or need health care in the past two (2) days, please answer for the last few times you did.

12. Over the past two days (48 hours) I agreed with the way decisions were made for _____:

not at all 0 1 2 3 4 5 6 7 8 9 10 completely

13. Over the past two days (48 hours) the quality of health care we received was:

unsatisfactory 0 1 2 3 4 5 6 7 8 9 10 extremely good

14. Over the past two days (48 hours) I felt my interaction with _____ was:

very comfortable 0 1 2 3 4 5 6 7 8 9 10 stressful

15. Over the past two days (48 hours), overall, I felt my interactions with the other people most important to me were:

very comfortable 0 1 2 3 4 5 6 7 8 9 10 stressful

16. Over the past two days (48 hours) my financial situation has been stressful:

not at all 0 1 2 3 4 5 6 7 8 9 10 completely

Appendix D

Caregiver Reaction Assessment

I would like you to rate the perceived impact of caregiving for each item on a five point scale, suing the following: **1 = strongly disagree 2 = disagree 3 = do not agree nor disagree 4 = agree 5 = strongly agree**

---

1. My activities are centered on care for my partner/family member	1	2	3	4	5
2. I am healthy enough to care for my partner/family member	1	2	3	4	5
3. My family works together at caring for my partner/family member	1	2	3	4	5
4. Caring for my partner/family member is important to me	1	2	3	4	5
5. It takes all my physical strength to care for my partner/family member	1	2	3	4	5
6. I enjoy caring for my partner/family member	1	2	3	4	5
7. I have to stop in the middle of my work or activities to provide care	1	2	3	4	5
8. My health has gotten worse since I've been caring for my partner/family member.	1	2	3	4	5
9. Since caring for my partner/family member, I feel my family has abandoned me.	1	2	3	4	5
10. Caring for my partner/family member makes me feel good	1	2	3	4	5
11. It is difficult to get help from my family in taking care of my partner/family member.	1	2	3	4	5
12. I feel privileged to care for my partner/family member.	1	2	3	4	5
13. Others have dumped caring for my partner/family member onto me.	1	2	3	4	5
14. I have eliminated things from my schedule since caring for my partner/family member.	1	2	3	4	5
15. I resent having to care for my partner/family member.	1	2	3	4	5

Caregiver Reaction Assessment Tool (cont)

1 = strongly disagree 2 = disagree 3 = do not agree nor disagree 4 = agree 5 = strongly agree

---

16. The constant interruptions make it difficult to find time for relaxation.	1	2	3	4	5
17. My family (brothers, sisters, children) left me alone to care for my partner/family member.	1	2	3	4	5
18. Since caring for my partner/family member, it seems like I'm tired all of the time.	1	2	3	4	5
19. I really want to care for my partner/family member.	1	2	3	4	5
20. I visit family and friends less since I have been caring for my partner/family member.	1	2	3	4	5
21. I will never be able to do enough caregiving to repay my partner/family member.	1	2	3	4	5
22. Financial resources are adequate.	1	2	3	4	5
23. It is difficult to pay for my partner/family member.	1	2	3	4	5
24. Caring for my partner/family member puts a financial strain on me.	1	2	3	4	5

Appendix E
Note templates for MD/ NP Palliative Care intervention
(based on note template from Cancer Support Team)

Usual Medical Note with adjustment to Assessment and Recommendation Sections—

Initial Consultation Note:
CC:
History of present illness (HPI):
Past medical history (PMH):
ADR:
Medications:
Family History:
Social History:
Review of systems (ROS):
Physical exam (PE):
Labs/ Imaging:
Assessment/ Recommendations:

- Symptoms:
    1.
    2.
    3.
    etc
- Psychosocial Issues:
- Spiritual Care:
- Advanced Care Planning:
- Caregiver Wellbeing/concerns:
- Referral to other specialties or other services
- Length of visit: xx minutes were spent with patient.

Follow-Up Note:
CC:
HPI:
ADR:
Meds:
ROS:
PE:
Labs/ Imaging:
Assessment/ Recommendations:

- Symptoms:
    1.
    2.
    3.
    etc
- Psychosocial Issues:
- Spiritual Care:
- Advanced Care Planning:

Caregiver Wellbeing/concerns:
Referral to other specialties or other services
Length of visit: xx minutes were spent with patient.

Appendix F
Note templates for Psychosocial Intervention
(interventions by social worker, Psych CNS, and Spiritual Care)
Notes involving the caregiver will be entered in the patient chart under the patient MRN.

Social work note:
This is #### (an in person visit, a telephone communication) with the #### (patient, patient caregiver) participating in the clinical trial CASE 5Y15. #### was (randomized to supportive care arm of study or referred by: Palliative care MD, palliative care NP, primary oncologist).

Brief summary discussion

Assessment/plan:
Living situation:
Coping/distress:
Mental health:
Relationships/Support:
Spiritual care:
Finances:
Referrals made for: home care services / psychological support / the gathering place / support group / spiritual care / educations resources / etc.
Time spent: #### minutes were spent with patient/caregiver

Mental health specialist note:
This is #### (an in person visit, a telephone communication) with the #### (patient, patient caregiver) participating in the clinical trial CASE 5Y15. #### was (randomized to supportive care arm of study or referred by: Palliative care MD, palliative care NP, primary oncologist).

Brief summary of discussion

Assessment/plan:
Anxiety surrounding diagnosis/treatment:
Mortality/ Death and dying:
Family relationships/communication:
Other:
Time spent:

Spiritual care note:

This is #### (an in person visit, a telephone communication) with the #### (patient, patient caregiver) participating in the clinical trial CASE 5Y15. #### was (randomized to supportive care arm of study or referred by: Palliative care MD, palliative care NP, primary oncologist).

Brief summary of discussion

Assessment/plan:
Sources of strength:
Sources of challenge:
Life purpose/meaning:
Relationship/support:
Time spent:

Phone note:
(patient or caregiver) called for ####. (If caregiver, please specify if calling for self or for patient)
Brief summary of discussion
Call referred to RN or MD: yes/no/NA
Was appointment made: yes/no
Time spent: #### minutes spent

Email to Primary Oncology Attending
Dear Dr. ****,
I saw (PT NAME AND MRN) on (DATE) for a palliative care intervention as part of the study ****. During this visit I addressed the following issues with the patient and his/her family:
Symptoms:
Psychosocial:
Spiritual Care:
Advanced Care Planning:
Caregiver Wellbeing:
Please see my note dated **** for details of the plan. If you have any questions or concerns please feel free to contact me at ****.

Appendix G: Radomization Key

Subject Number	Random Permutation	Treatment Assignment
1501001	4	Structured Palliative Care
1501002	3	Structured Palliative Care
1501003	1	Standard care
1501004	2	Standard care
1501005	3	Structured Palliative Care
1501006	1	Standard care
1501007	4	Structured Palliative Care
1501008	2	Standard care
1501009	2	Standard care
1501010	4	Structured Palliative Care
1501011	1	Standard care
1501012	3	Structured Palliative Care
1501013	3	Structured Palliative Care
1501014	2	Standard care
1501015	1	Standard care
1501016	4	Structured Palliative Care
1501017	4	Structured Palliative Care
1501018	2	Standard care
1501019	1	Standard care
1501020	3	Structured Palliative Care
1501021	4	Structured Palliative Care
1501022	2	Standard care
1501023	3	Structured Palliative Care
1501024	1	Standard care
1501025	3	Structured Palliative Care
1501026	4	Structured Palliative Care
1501027	1	Standard care
1501028	2	Standard care
1501029	2	Standard care
1501030	1	Standard care
1501031	4	Structured Palliative Care
1501032	3	Structured Palliative Care
1501033	3	Structured Palliative Care
1501034	1	Standard care
1501035	2	Standard care
1501036	4	Structured Palliative Care
1501037	2	Standard care
1501038	3	Structured Palliative Care
1501039	4	Structured Palliative Care
1501040	1	Standard care
1501041	3	Structured Palliative Care

1501042	4	Structured Palliative Care
1501043	2	Standard care
1501044	1	Standard care
1501045	1	Standard care
1501046	2	Standard care
1501047	3	Structured Palliative Care
1501048	4	Structured Palliative Care
1501049	1	Standard care
1501050	2	Standard care
1501051	3	Structured Palliative Care
1501052	4	Structured Palliative Care
1501053	2	Standard care
1501054	1	Standard care
1501055	4	Structured Palliative Care
1501056	3	Structured Palliative Care
1501057	3	Structured Palliative Care
1501058	2	Standard care
1501059	1	Standard care
1501060	4	Structured Palliative Care
1501061	3	Structured Palliative Care
1501062	2	Standard care
1501063	4	Structured Palliative Care
1501064	1	Standard care
1501065	3	Structured Palliative Care
1501066	4	Structured Palliative Care
1501067	1	Standard care
1501068	2	Standard care
1501069	2	Standard care
1501070	3	Structured Palliative Care
1501071	1	Standard care
1501072	4	Structured Palliative Care
1501073	2	Standard care
1501074	3	Structured Palliative Care
1501075	4	Structured Palliative Care
1501076	1	Standard care
1501077	4	Structured Palliative Care
1501078	1	Standard care
1501079	3	Structured Palliative Care
1501080	2	Standard care

Appendix H

Acrobat Document
